# CPEB1-dependent disruption of the mRNA translation program in oocytes during maternal aging

**DOI:** 10.1038/s41467-023-35994-3

**Published:** 2023-01-26

**Authors:** Nozomi Takahashi, Federica Franciosi, Enrico Maria Daldello, Xuan G. Luong, Peter Althoff, Xiaotian Wang, Marco Conti

**Affiliations:** 1 Center for Reproductive Sciences, University of California, San Francisco, CA 94143 USA; 2 USA Eli and Edythe Broad Center of Regeneration Medicine and Stem Cell Research, University of California, San Francisco, CA 94143 USA; 3 Department of Obstetrics and Gynecology and Reproductive Sciences, University of California, San Francisco, CA 94143 USA; 4 Present Address: Reproductive and Developmental Biology Lab, Department of Veterinary Medicine and Animal Science, Università degli Studi di Milano, 20133 Milan, Italy; 5 Present Address: Sorbonne Université, CNRS, Laboratoire de Biologie du Développement—Institut de Biologie Paris Seine, LBD-IBPS, Paris, France

**Keywords:** Translation, Ageing

## Abstract

The molecular causes of deteriorating oocyte quality during aging are poorly defined. Since oocyte developmental competence relies on post-transcriptional regulations, we tested whether defective mRNA translation contributes to this decline in quality. Disruption in ribosome loading on maternal transcripts is present in old oocytes. Using a candidate approach, we detect altered translation of 3’-UTR-reporters and altered poly(A) length of the endogenous mRNAs. mRNA polyadenylation depends on the cytoplasmic polyadenylation binding protein 1 (CPEB1). *Cpeb1* mRNA translation and protein levels are decreased in old oocytes. This decrease causes de-repression of *Ccnb1* translation in quiescent oocytes, premature CDK1 activation, and accelerated reentry into meiosis. De-repression of *Ccnb1* is corrected by *Cpeb1* mRNA injection in old oocytes. Oocyte-specific *Cpeb1* haploinsufficiency in young oocytes recapitulates all the translation phenotypes of old oocytes. These findings demonstrate that a dysfunction in the oocyte translation program is associated with the decline in oocyte quality during aging.

## Abstract (web summary)

In the absence of transcription, gene expression required for oocyte maturation is regulated by translation of preexisting mRNAs. Here the authors show that a defective program of mRNA translation in oocyte is associated with maternal aging.

## Introduction

Loss of homeostasis and decreased ability to respond to metabolic challenges is a hallmark of cellular aging^1^. However, the aging-related decline in cellular functions does not progress in a synchronous fashion in all cells and tissues of the body. One striking example is the ovary, where an age-related decrease in the number and quality of oocytes causes a decline in fertility that precedes other aging-induced loss of function^2^. Although aging patients can carry a pregnancy to term with oocyte donation^3,4^, the decrease in oocyte developmental competence is a major hurdle in the Assisted Reproductive Technologies (ARTs) setting. The decline in oocyte quality has been imputed to several causes, with an increased incidence of aneuploidy of the egg or the embryo and genomic instability perhaps caused by epigenetic errors or metabolic insults being the most widely reported^5–7^.

The oocyte’s ability to develop as an embryo and to support pregnancy through a live birth relies on a complex set of developmental processes, including the coordinated acquisition of gamete meiotic and cytoplasmic competence^8,9^. Less clear is the role of cytoplasmic competence in the decline in fertility with age. Cytoplasmic maturation includes the assembly of the molecular machinery necessary for the completion of meiosis, for reprogramming of organelle morphology and their redistribution within the oocyte. It should be underscored that upon fertilization the oocyte cytoplasm is transferred to the zygote. Therefore, the transcriptional activation of the embryo genome takes place in this maternal environment^9–11^. Several observations in the ART practice point to defective cytoplasmic competence as a cause of the decrease in embryo fitness to develop and implant^12^.

Oocyte maturation is the last step of oogenesis. In a span of several hours, a Prophase I arrested oocyte (also called germinal vesicle (GV) stage oocyte) re-enters meiosis in response to the luteinizing hormone (LH) surge, undergoes nuclear envelop breakdown (NEBD or germinal vesicle breakdown, GVBD), completes the first meiotic division, and finally arrests at the MII stage of meiosis. MII oocytes are then ovulated and ready to be fertilized. Removal of a GV-arrested, fully grown oocyte, from the antral follicle initiates a cascade of events called spontaneous maturation that recapitulates maturation to the MII stage in vitro. During these final stages of oocyte maturation, the synthesis of proteins critical for developmental competence relies on a program of timed translation of long-lived mRNAs that have been synthesized earlier during oocyte growth^13–15^. Thus, gene expression in oocytes is controlled by translation in the cytoplasm rather than via transcription in the nucleus. This property, shared by the gametes of most species^16^, clearly indicates that the translational program executed at the oocyte-to-zygote transition is integral to the acquisition of developmental competence. We have provided evidence that defects in the translation program are associated with compromised developmental competence in the mouse^17–19^. One central mechanism of translation regulation of maternal mRNAs relies on the dynamic modulation of the poly(A) tail associated with the mRNA 3’-untranslated region (3’-UTR)^20^. A major regulator of polyadenylation is the cytoplasmic polyadenylation element binding protein 1 (CPEB1)^20,21^. This RNA-binding protein modulates the translation of mRNAs containing the cytoplasmic polyadenylation element (CPE). Our genome-wide analysis revealed a global switch in maternal mRNA translation coinciding with oocyte reentry into the meiotic cell cycle and the crucial role of CPEB1 in directing this switch^15^.

Here, we propose the hypothesis that a defective execution of the maternal mRNA translation program during oocyte maturation also leads to the compromised developmental competence observed during maternal aging. In contrast to the numerous transcriptomic studies published on oocyte aging^22–25^, scarce information is available as to whether defects in the translation program contribute to the age-related decline in oocyte quality^26,27^. Using a RiboTag Immunoprecipitation (IP) strategy^28^ in mouse oocytes^29^, we have compared ribosome loading in MII oocytes from young and old mice. We provide evidence for defects in the mRNA translation program in oocytes derived from old female mice, a disruption that is associated with decreased CPEB1 function. In turn, these defects likely contribute to the decline in developmental competence and age-associated infertility.

## Results

### RiboTag IP/RNA-Seq reveals a disruption in the pattern of mRNA translation during maternal aging

Previous studies have examined the transcriptome in quiescent (GV) and matured (MII) oocytes from young and old mice (dataset GDS3295)^24^. A conclusion of this study is that a significant number of transcripts are decreased in aging oocytes^24^. However, our re-analysis of these datasets also shows that levels of 2109 transcripts increase significantly from GV to MII in young oocytes (Supplementary Fig. 1a). Mouse oocyte maturation occurs in the virtual absence of transcription, as fully matured oocytes show highly condensed chromatin, and RNA synthesis is at background level^13^. Given this genome-wide transcriptional silencing, mRNA levels in maturing oocytes should only remain stable or decrease, the decrease being caused by destabilization/degradation^15,30^. Therefore, we hypothesize that the widespread increase in transcript levels is due to a bias towards polyadenylated mRNA species introduced during cDNA synthesis using oligo(-dT) primers, a possibility also raised in the original publication^24^. The apparent increase in mRNA levels would then reflect differences in polyadenylation/translation rather than mRNA steady-state levels, a possibility also proposed in other oocyte transcriptomic studies^31,32^. Indeed, a comparison of apparently increased transcripts in GDS3295^24^ with our datasets probing ribosome loading^14^ show considerable overlap with activated mRNAs (Supplementary Fig. 1a–c). Moreover, direct measurement of poly(A) length and efficiency of oligo(-dT) priming confirms such a correlation (Supplementary Fig. 2). The apparent differences between young and old oocytes found in the aging transcriptome may, therefore, indicate defective polyadenylation (Supplementary Fig. 1d) rather than altered transcript levels.

**Fig. 1:**
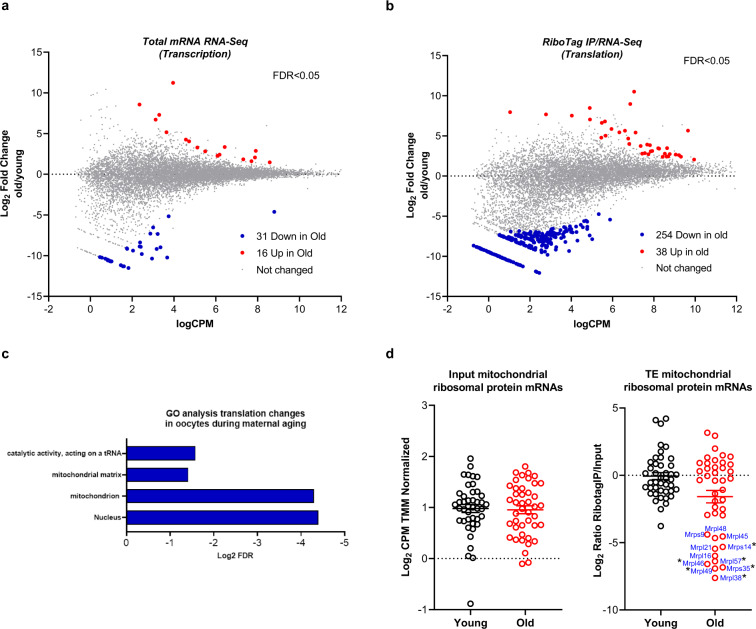
Differences in transcriptome and translatome in young and old MII oocytes. MII oocytes were collected from young and old *RiboTag*^*F/F*^*;Zp3-Cre* mice and RiboTag IP followed by RNA-Seq was performed as described in “Methods”. Two biological independent samples are used. **a**, **b** MA-plot of gene expression changes for total transcripts (**a**) and ribosome loading on transcripts (**b**) are reported. The log_2_ fold changes for young vs old oocytes is plotted on the *y* axis and the log_10_ of the average counts per million reads (CPM) is shown on the *x* axis. Each gene is represented by a dot. Genes with FDR < 0.05 are shown in red (upregulated) or blue (downregulated). **c** Gene ontology analysis of mRNAs whose translation is significantly (FDR < 0.1) different between young and old oocytes, including both upregulated and downregulated transcripts. Note the highly significant enrichment in mitochondrion term. **d** Comparison of levels of mRNAs coding for mitochondrial ribosomal protein and their translation efficiency (TE) in young and old oocytes. The log_2_ of the average CPM is plotted on the *y* axis. The bar represents mean $\pm$ SEM. A subset of mRNAs coding for mitochondrial ribosomal proteins is poorly translated in aging oocytes. *FDR < 0.05. Source data are provided as a Source Data file.

To directly assess this possibility, we compared transcriptome and translatome in MII oocytes from young (1 month) and old (12–15 months) mice using the RiboTag IP/RNA-Seq ribosome-loading strategy^29^ and random priming library preparation. Properties of the oocyte samples are reported in Supplementary Fig. 3a–e, and data quality control analysis of the RNA-Seq data is reported in Supplementary Fig. 4a, b. With the exception of 47 mRNAs, total mRNA levels (input) did not differ significantly between young and old MII oocytes (FDR < 0.05, Fig. 1a). Conversely, ribosome loading was significantly decreased for 254 mRNAs and increased for 38 mRNAs in old oocytes compared with young oocytes (FDR < 0.05. Fig. 1b). The list of input and HA genes significantly different are provided in Supplementary Data 1 and 2. Relaxing the statistical stringency to FDR < 0.1 increased the number of mRNAs with significantly altered ribosome loading to 726. Gene ontology analysis of the decreased and increased mRNAs showed enrichment in terms that include “mitochondrion” and “nucleus” (Fig. 1c). Further mining of the data showed that the translation efficiency for a significant number of mRNAs encoding mitochondrial ribosomal proteins is disrupted in old oocytes compared to young, although input levels are comparable (Fig. 1d). Alteration in mitochondrial ribosomal protein mRNA translation has been reported for several tissues of an aging organism^33–35^, and mitochondrion dysfunction during aging is well-established^36^ including for the oocytes^37^. Thus, our RiboTag IP/RNA-Seq analysis detected changes shown to be associated with aging and provided an indication that disruption of mRNA translation is present in old oocytes.

### 3’-UTRs of affected transcripts drive defective mRNA translation during maternal aging

For a better characterization of the defects in translation associated with maternal aging, we used a candidate gene approach. To capture the broad changes suggested in the previous aging transcriptome and detected by RNA-Seq, we included the following genes among the candidates analyzed. TPX2 was used as a control, not being significantly different in the aging transcriptome and in our RNA-Seq. NLRP5 (MATER), a component of the subcortical maternal complex^38^ for which an increase was detected in the RNA-Seq and in the aging transcriptome, was used as a prototypic upregulated mRNA during aging. *Golph3*, *Il7*, and *Thumpd1* were used as representative downregulated mRNAs, with a decrease detected in one or both datasets. GOLPH3 is a component of the Golgi apparatus, an organelle undergoing major restructuring during oocyte maturation^39^. THUMPD1 encodes an RNA-binding protein involved in the acetylation of RNA and ribosome biogenesis^40^. We have previously shown that translation of IL7 mRNA and protein secretion increase during oocyte maturation, and its accumulation in follicular fluid (FF) of in vitro fertilization (IVF) patients correlates with oocyte maturation and egg quality^41^. Given this correlation between IL7 secretion and oocyte quality, we included this candidate because it is significantly decreased in the aging transcriptome, even though no changes in translation were detected for this mRNA in the RNA-Seq dataset.

**Fig. 2:**
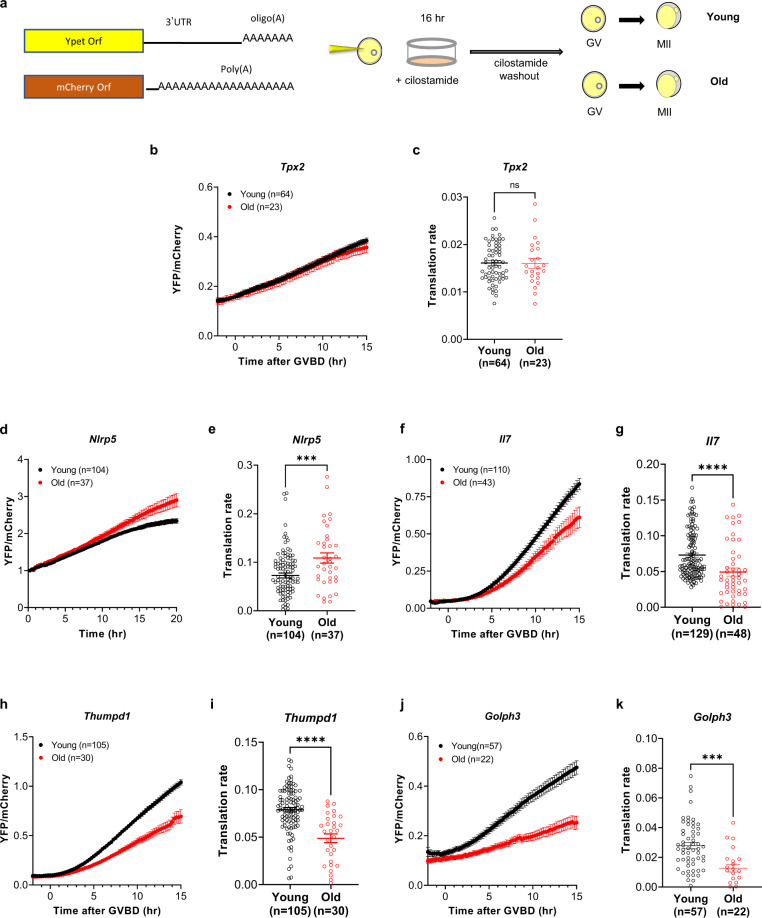
Complex alterations in translation are detected in old oocytes compared to young oocytes. **a** Scheme of YFP reporter assay. GV oocytes from young and old mice were injected with 12.5 ng/μl polyadenylated mCherry and 12.5 ng/μl of oligoadenylated YFP reporters, including *Tpx2, Nlrp5, Il7, Thumpd1,* and *Golph3* 3’-UTR. After overnight pre-incubation, oocytes were released in cilostamide-free medium, and YFP and mCherry signals were recorded by time-lapse microscopy every 15 min for 20 h during maturation. The YFP signals were normalized by the level of mCherry signals. **b**, **d**, **f**, **h**, **j** The ratio of YFP/mCherry signal for each oocyte was plotted according to the incubation time (**d**) or with time of GVBD set as 0 time (**b**, **f**, **h**, **j**). **c**, **e**, **g**, **i**, **k** Translation rates were calculated for each oocyte by linear regression of the reporter data between 5 and 10 h (**b**, **j**), between 7 and 12 h after GVBD (**f**, **h**), or between 15 and 20 h after incubation time (**d**). Experiments were repeated three times with different batches of oocytes from different cohorts of aging mice and the data are shown as the mean $\pm$ SEM. Two-tailed unpaired Student’s *t* tests were used to evaluate statistical significance, ns not significant, **e** ***P = 0.0008, **g** ****P < 0.0001, **h** ****P < 0.0001, **k** ***P = 0.0002. Source data are provided as a Source Data file.

Reporters were generated by fusing the enhanced YFP fluorescent protein to the 3’-UTR of *Tpx2*, *Nlrp5*, *Golph3*, *Il7* or *Thumpd1* mRNAs. After in vitro translation, these construct mRNAs were injected together with an mCherry mRNA in denuded oocytes from young and old mice, and the rate of translation was monitored by time-lapse microscopy throughout maturation (Fig. 2a). Used as a loading control, mCherry signal was comparable for all reporters between young and old oocytes (Supplementary Fig. 5). Translation pattern can be divided into three main categories. In addition to a pattern of constitutive translation, mRNAs undergo decreased translation during oocyte maturation as *Nlrp5*, while activated translation is observed for another group, including *Il7*, *Thumpd1*, and *Golph3*. Supplementary Fig. 6 highlights two opposing patterns of ribosome loading during maturation and YFP accumulation in oocytes maintained in GV or during in vitro maturation. *Nlrp5* translation in young oocytes progressively decreased to a plateau (Supplementary Fig. 6c and Fig. 2d), reflecting repression of translation, a finding consistent with ribosome loading (Supplementary Figs. 1c and 6a). However, in old oocytes the rate of translation of this reporter was initially comparable in the two groups but, with time, it did not plateau as in young oocytes (Fig. 2d). This suggests that translation in old oocytes is not correctly repressed, a finding consistent with the data from the aging transcriptome and ribosome loading. The YFP accumulation driven by the *Golph3*, *Il7*, and *Thumpd1* 3’-UTR underwent activation about 3 h after GVBD and continued to increase thereafter (Supplementary Fig. 6d and Fig. 2f, h, j), findings again consistent with the ribosome loading (see supplementary Figs. 1c and 6b). However, a decreased accumulation of the reporter was detected in old oocytes for all three 3'-UTRs. To quantify differences in reporter translation, the rate of accumulation was calculated for each injected oocyte using curve fitting^42^. Using this approach, the translation rate of *Golph3*, *Il7,* and *Thumpd1* is significantly decreased in old oocytes compared to young (Fig. 2g, i, k), while the translation of *Nlrp5* is significantly increased in old oocytes (Fig. 2e). No difference in translation was detected in *Tpx2* (Fig. 2b, c), confirming that altered translation is not a generalized phenomenon. Therefore, the increased, decreased, or unchanged accumulation of these reporters is consistent with the apparent changes in the aging transcriptome and with our ribosome loading data.

Given our previous finding that translation in oocytes is sensitive to the presence of surrounding cumulus cells^17^, we then tested whether translation under these more physiological conditions were altered in old oocytes. When oocytes are still surrounded by the cumulus cells, they are difficult to monitor via time-lapse microscopy due to movement as the cumulus expands. Therefore, we used Renilla luciferase (RL) reporters fused with the 3’-UTR of target mRNA injected together with firefly luciferase (FL) into oocytes while still surrounded by cumulus cells (COCs) (Supplementary Fig. 7a). At the end of the incubation, oocytes were denuded, lysed, and used for luciferase assay. In all cases, the translation of reporters was increased from GV to MII (Supplementary Fig. 7b–d). In agreement with the denuded oocyte data, translation of *Il7* and *Thumpd1* was again significantly decreased in old MII oocytes compared with young MII oocytes (Supplementary Fig. 7c, d), whereas no difference in *Tpx2* translation was detected (Supplementary Fig. 7b). Thus, maintaining somatic/germ cell interaction does not rescue the defective translation.

### Endogenous polyadenylation of mRNAs is decreased in old oocytes

**Fig. 3:**
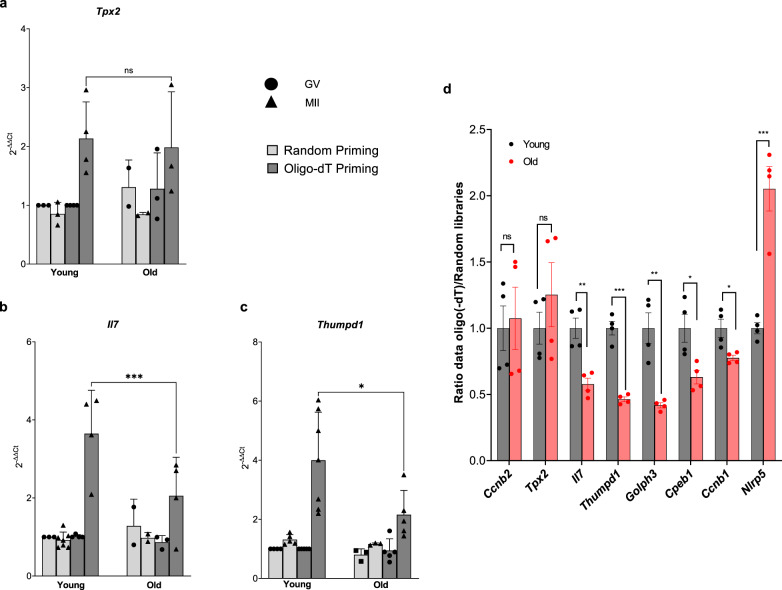
Endogenous mRNA Polyadenylations are altered with maternal aging. **a**–**c** RNA of GV or MII oocytes from young or old mice was reverse-transcribed using random hexamer or oligo(-dT) primers, and quantitative PCR was performed with gene-specific primers. Data are normalized by the average expression of *Dppa3*, *Rpl19*, *ActB*, and *Eif4a1* as detailed in “Methods”. **a** *n* = 3 (GV young random), 3 (MII young random), 4 (GV young oligo(-dT)), 4 (MII young oligo(-dT)), 2 (GV old random), 2 (MII old random), 3 (GV old oligo(-dT)), 3 (MII old oligo(-dT)) biologically independent samples are used. **b** *n* = 3 (GV young random), 7 (MII young random), 4 (GV young oligo(-dT)), 4 (MII young oligo(-dT)), 2 (GV old random), 2 (MII old random), 3 (GV old oligo(-dT)), 4 (MII old oligo(-dT)) biologically independent samples are used. **c** *n* = 4 (GV young random), 5 (MII young random), 5 (GV young oligo(-dT)), and 7 (MII young oligo(-dT)), 3 (GV old random), 3 (MII old random), 5 (GV old oligo(-dT)), 5 (MII old oligo(-dT)) biologically independent samples are used. The data are shown as the mean $\pm$ SD. Two-tailed paired Student’s *t* tests were used to evaluate statistical significance, ns not significant, *P = 0.029, ***P = 0.0003. **d** The ratio of data from oligo(-dT)^24^ and random primed RNA-Seq libraries between young and old oocytes is reported. Four biologically independent oligo(-dT) samples and two biologically independent random primed samples are used. The data are shown as the mean $\pm$ SD. A significantly decreased ratio is detected for *Il7*, *Thumpd1*, *Ccnb1*, *Golph3*, and *Cpeb1* mRNAs. Conversely, putative changes polyadenylation of *Ccnb2* and *Tpx2* mRNAs is not affected. Two-tailed multiple unpaired *t* tests were used to evaluate statistical significance, ns not significant, **P = 0.0032 for *Il7*, ***P = 0.000061 for *Thumpd1*, **P = 0.0025 for *Golph3*, *P = 0.019 for *Cpeb1*, *P = 0020 for *Ccnb1*, ***P = 0.00087 for *Nlrp5*. Source data are provided as a Source Data file.

In oocytes, activation of translation is dependent on the elongation of the poly(A) tail of an mRNA. To assess this property, we compared the polyadenylation state of the endogenous *Tpx2*, *Il7*, and *Thumpd1* mRNAs in young and old oocytes using a strategy where qPCR signals from cDNA synthesized by either oligo(-dT) or random priming are compared. This is a widely employed assay when there are a limited number of cells available^43^. As reported in Fig. 3a–c, the three transcripts show an apparent increase in MII versus GV with oligo(-dT) priming, whereas no significant changes were observed with random priming. This is consistent with the hypothesis that the polyadenylation of the three transcripts increases during oocyte maturation. More importantly, when comparing young and old oocytes, the signals from oligo(-dT) priming were significantly decreased for both *Il7* and *Thumpd1* mRNAs (Fig. 3b, c), whereas *Tpx2* polyadenylation was again unchanged (Fig. 3a). These data indicate that endogenous polyadenylation is defective in old oocytes, a finding consistent with the decreased translation detected with the reporter assays. To extend the correlation oligo(-dT)/random priming to additional mRNAs, we compared the deposited MII data obtained with oligo(-dT) priming and our random priming dataset. The ratio of oligo(-dT)/random priming is comparable in young and old MII samples for most of the genes. However, significant differences in the ratio were detected for a subgroup of transcripts. These include the transcripts coding for *Il7, Thumpd1, Golph3*, and *Nlrp5*. Also, changes were detected for *Cpeb1* and *Ccnb1* (see below), while no differences in the ratio were observed for *Tpx2* and *Ccnb2* (Fig. 3d). Thus, these altered ratios are consistent with defective mRNA polyadenylation during aging.

### Translation of CPEB1 is decreased in old oocytes

**Fig. 4:**
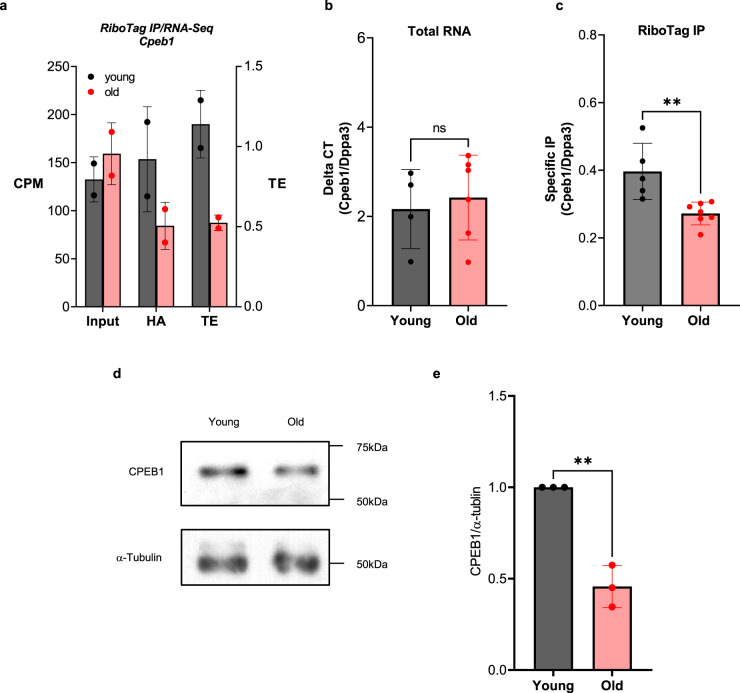
Translation of CPEB1 mRNA and protein is decreased in old oocytes. **a** MII oocytes were collected from young and old *RiboTag*^*F/F*^*;Zp3-Cre* mice and RiboTag IP followed by RNA-Seq was performed as described in “Methods”. The average CPM for total transcripts (Input) and ribosome loading of transcripts (HA) are reported. TE was calculated as the ratio between ribosome-associated and total mRNA CPM. Two independent biological samples were used in this analysis. **b**, **c** Ovaries were collected from young and old *RiboTag*^*F/F*^*;Zp3-Cre* mice and RiboTag IP followed by RT-qPCR was performed as described in “Methods”. *Dppa3* was used as a reference gene. Input data (**b**) and ribosome-loading mRNA (**c**) are reported. **b** *n* = 4 (young), 6 (old) biologically independent samples are used. **c** *n* = 5 (young), 7 (old) biologically independent samples are used. RT-qPCR reactions were run in triplicate. The bars represent the mean ± SD. Two-tailed unpaired Student’s *t* tests were used to evaluate statistical significance, ns not significant, **P = 0.0048. **d**, **e** CPEB1 protein expression in GV oocytes from young and old mice. Accumulation of α-tubulin was used as a control. Oocytes were lysed in sample buffer and used for western blot analysis. Twenty-five oocytes per lane were loaded for the experiment. A representative of three experiments is reported. A graph shows quantification of a western blot on three independent biological replicates. The data are reported as the mean $\pm$ SD. CPEB1/α-tubulin ratios were expressed as fold changes over young controls. Two-tailed unpaired Student’s *t* tests were used to evaluate statistical significance, **P = 0.0012. Source data are provided as a Source Data file.

Polyadenylation of mRNAs and their translation during oocyte maturation, in large part, depends on the function of the RNA-binding protein (RBP) CPEB1^20^. This RBP interacts with *cis*-acting elements located in the 3’-UTR of an mRNA and assembles a complex that promotes adenylation. Putative CPEs were identified in *Il7, Thumpd1*, and *Golph3* mRNAs, but not in *Nlrp5* mRNAs. Moreover, RNA-IP with CPEB1 antibodies conclusively confirms specific CPEB1-binding to endogenous *Il7, Thumpd1*, and *Golph3* mRNAs in the oocyte (Supplementary Fig. 8). Therefore, we hypothesized that a defective function of CPEB1 may be a cause of decreased polyadenylation and translation of these mRNAs in aging oocytes. Indeed, the RiboTag IP/RNA-Seq data indicate that, although not reaching statistical significance, the translation efficiency (TE)^44^, defined as the ratio between ribosome-bound and total mRNA levels, for the *Cpeb1* mRNA is decreased ~50% in oocytes during maternal aging, whereas total mRNA levels are comparable between young and old oocytes (Fig. 4a). To confirm this observation, we performed RiboTag IP/qPCR using ovary extracts from young and old mice. Since the Cre recombinase in the RiboTag mouse is driven by the ZP3 promoter, the RiboTag IP of total ovary extract probes only translation in oocytes from secondary to antral follicles. Using this approach, total *Cpeb1* mRNA (input) did not differ between young and old ovaries (Fig. 4b); however, ribosome-bound mRNA was significantly decreased in ovaries from old mice (Fig. 4c) (*P* < 0.01). Decreased translation of the *Cpeb1* mRNA was further confirmed by a western blot of oocytes from young and old mice. CPEB1 protein levels are significantly decreased by ~50% in old oocytes compared to young (Fig. 4d, e). Of note, an “apparent” and significant decrease in CPEB1 levels is found in old oocytes with the aging transcriptome (Supplementary Fig. 1d) as well as an apparent decrease in polyadenylation (Fig. 3d). These results suggest that the decrease in CPEB1 protein expression in old oocytes is due to decreased translation throughout the growing follicle stages as well as during maturation.

### An accelerated meiosis reentry in old oocytes is driven by increased Ccnb1 translation

**Fig. 5:**
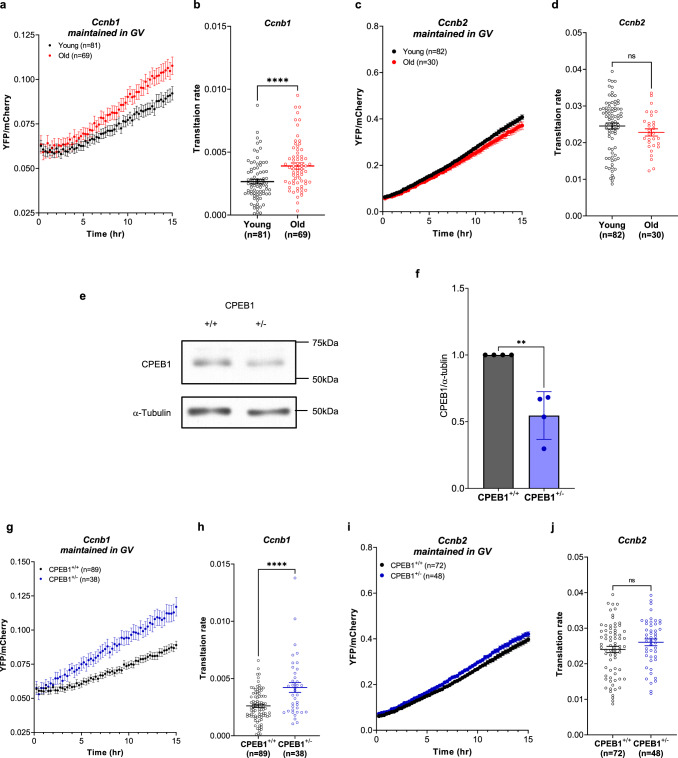
Decreased CPEB1 expression de-represses Ccnb1 translation during the GV stage. **a**–**d, g**–**j** GV oocytes from young and old mice (**a**–**d**) or *Cpeb1*^*+/+*^*;Zp3Cre* mice (CPEB1^+/+^ oocytes) and *Cpeb1*^*fl/+*^*;Zp3Cre* mice (CPEB1^+/−^ oocytes) (**g**–**j**) were injected with polyadenylated mCherry and *YFP-Ccnb1* 3’-UTR (**a**, **b**, **g**, **h**) or *YFP-Ccnb2* 3-’UTR (**c**, **d**, **i**, **j**). After 3 h of pre-incubation, oocytes were maintained in GV stage with cilostamide treatment. YFP and mCherry signals were recorded by time-lapse microscopy every 15 min for 15 h. The YFP signals were normalized by the level of mCherry signals. **a**, **c**, **g**, **i** YFP/mCherry signal in each oocyte was plotted depending on the incubation time. **b**, **d**, **h**, **j** Translation rate was calculated for each oocyte by linear regression of the reporter data between 3 and 13 h. Experiments were repeated three times and the data are shown as the mean $\pm$ SEM. Two-tailed unpaired Student’s *t* tests were used to evaluate statistical significance, ns not significant, ****P < 0.0001. **e**, **f** CPEB1 protein expression in CPEB1^+/+^ GV oocytes and CPEB1^+/−^ GV oocytes. Accumulation of α-tubulin was used as a control. Oocytes were lysed in sample buffer and used for western blot analysis with 30 oocytes per lane loaded for the experiment. **e** A representative of four experiments is reported. **f** A graph shows the quantification of four independent western blot analyses. The bar represents mean $\pm$ SD. CPEB1/α-tubulin ratios were expressed as fold changes over CPEB1^+/+^ oocytes. Two-tailed unpaired Student’s *t* tests were used to evaluate statistical significance, **P = 0.0023. Source data are provided as a Source Data file.

**Fig. 6:**
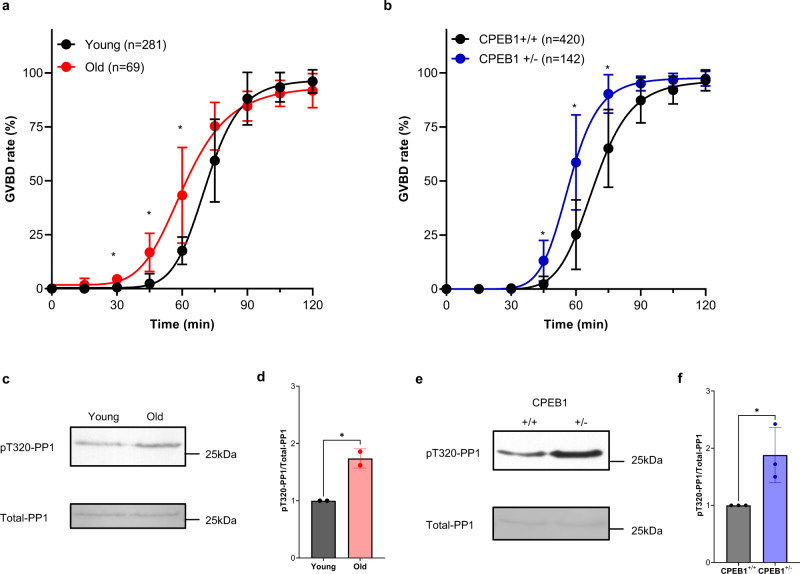
Decreased CPEB1 expression accelerates meiosis reentry. **a**, **b** GV oocytes from young and old mice (**a**), or GV oocytes from *Cpeb1*^*+/+*^*;Zp3Cre* mice (CPEB1^+/+^ oocytes) and *Cpeb1*^*fl/+*^*;Zp3Cre* mice (CPEB1^+/−^ oocytes) (**b**) were incubated with cilostamide-free medium and brightfield images were captured every 15 min. The cumulative GVBD times were plotted. Experiments were repeated at least three times, and the data are shown as the mean $\pm$ SD. Two-tailed multiple unpaired Student’s *t* tests were used to evaluate statistical significance at each time point. **a** *P* = 0.00032 for 30 min, *P* = 0.0076 for 45 min, *P* = 0.016 for 60 min. **b** *P* = 0.0036 for 45 min, *P* = 0.0051 for 60 min, *P* = 0.019 for 75 min. **c**–**f** CDK1 activity were measured using Kinase assays. Young and old GV oocytes (**c**, **d**), or CPEB1^+/+^ and CPEB1^+/−^ GV oocytes (**e**, **f**) were incubated with cilostamide-free medium for 45 min. After oocytes were lysed by freezing and thawing, oocytes were incubated with GST-PP1 fragment as a substrate for 30 min. Oocytes were lysed in a sample buffer and used for western blot analysis. T320 PP1 phosphorylation was detected by a phospho-specific antibody (pT320-PP1). The level of total substrate loaded was evaluated by Ponceau S staining (Total PP1). Ten oocytes per lane were loaded for the experiment. **c**, **e** A representative of experiments is reported. **d**, **f** A graph shows the quantification of two or three independent western blot analyses. The data are shown as the mean $\pm$ SD. pT320-PP1/ total PP1 ratios were expressed as fold changes over young controls. Two-tailed unpaired Student’s *t* tests were used to evaluate statistical significance, **d** *P = 0.025, **f** *P = 0.033. Source data are provided as a Source Data file.

CPEB1 is a critical RBP with complex functions during oocyte development. It is also considered a major regulator of progression through the meiotic cell cycle, given its role in the regulation of translation of critical cell cycle regulators, including cyclins. If aging were associated with a dysfunction in CPEB1, then an altered translation pattern of the prototypic CPEB1 target *Ccnb1* would be expected. This possibility was verified by injection of a reporter driven by the 3’-UTR of *Ccnb1* in oocytes from young and old mice and probing its rate of translation during GV stage. In fully grown GV oocytes, CPEB1 functions as a repressor of translation. Indeed, we found that the rate of translation of the *Ccnb1* reporter is increased in GV oocytes from old mice as compared to those from young mice (Fig. 5a, b). As a negative control, we used the translation of another cyclin, *Ccnb2*, which we have shown not to be repressed by CPEB1 in GV oocytes^15,45^. No differences in the *Ccnb2* reporter signal were observed between young and old GV oocytes (Fig. 5c, d). Given the fact that CCNB1 functions as a regulatory subunit of CDK1, the master regulator of meiosis progression, we tested whether a dysfunction in CPEB1 associated with aging would have any effects on the timing of meiotic cell cycle progression. We incubated oocytes in a maturation medium and recorded the time of GVBD using time-lapse microscopy every 15 min. Under these conditions, reentry into meiosis is significantly accelerated by ~15 min in old oocytes (Fig. 6a). To verify a premature activation of CDK1, oocyte extracts were incubated with a CDK1 substrate protein phosphatase 1 (GST-PP1) peptide, and phosphorylation was measured by phospho-specific antibodies (pT320-PP1)^46–48^. Considering that the maximum difference in GVBD time between young and old oocytes is 60 min after incubation (Fig. 6a) and CDK1 activation occurs before GVBD, we examined CDK1 activity in young and old oocytes after 45 min of removal of the PDE inhibitor. CDK1 activity was significantly increased in old compared with young oocytes (Fig. 6c, d). These results indicate that a loss in translational repression by CPEB1 during Prophase I causes an increased accumulation of CCNB1, therefore affecting the threshold levels of CDK1 activity as well as the timing of meiotic reentry.

### Haploinsufficiency of CPEB1 in young mice recapitulates the translation and meiotic reentry phenotype of old oocytes

To test if haploinsufficiency of CPEB1 recapitulates the phenotypes associated with oocyte aging, we used conditional CPEB1 heterozygous (*Cpeb1*^fl/+^;*Zp3-Cre*) mice. Western blotting detected a 50% decrease in CPEB1 protein in young CPEB1^+/−^ oocytes in GV state (Fig. 5e, f). This decrease in CPEB1 expression is comparable to that observed in old mice (Fig. 4d, e). To examine whether this partial loss of CPEB1 in young oocytes recapitulates the phenotypes associated with aging, we examined translation rate of *Ccnb1*, GVBD time, and CDK1 activity in this quiescent state. These measurements in CPEB1^+/−^ oocytes show that the rate of translation of the *Ccnb1* reporter is increased in GV oocytes (Fig. 5g, h), whereas no differences were observed with the *Ccnb2* reporter (Fig. 5i, j). Accelerated GVBD time and increased CDK1 activity were also observed in CPEB1^+/−^ oocytes (Fig. 6b, e, f). Although differences are not major, a clear gene dosage-dependent effect was observed when comparing heterozygous and homozygous null for the *Cpeb1* locus (Supplementary Fig. 9a). Thus, genetic manipulation of CPEB1 levels in young oocytes completely recapitulates all the phenotypes that we have described in old oocytes.

### CPEB1 decreased function in old oocytes is rescued by injection of Cpeb1 mRNA

**Fig. 7:**
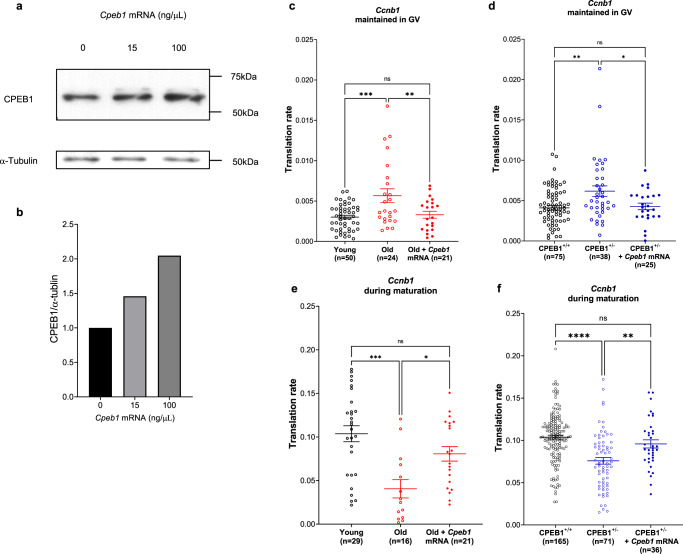
CPEB1 decreased function in old oocytes can be rescued by injection of Cpeb1 mRNA. **a**, **b** CPEB1 protein expression in GV oocytes injected with *Cpeb1* mRNA at different concentrations. Accumulation of the endogenous α-tubulin was used as a control. Oocytes were lysed in a sample buffer and used for western blot analysis. In total, 30 oocytes per lane were loaded for the experiment. **b** A graph shows quantification of a single western blot analysis. CPEB1/α-tubulin ratios were expressed as fold changes over control. **c**–**f** GV oocytes from young and old mice, or *Cpeb1*^*+/+*^*;Zp3Cre* mice (CPEB1^+/+^ oocytes) and *Cpeb1*^*fl/+*^*;Zp3Cre* mice (CPEB1^+/−^ oocytes) were injected with polyadenylated mCherry, *YFP-Ccnb1* 3’-UTR, and *Cpeb1* mRNA (100 ng/μl). After overnight pre-incubation, oocytes were maintained in GV stage with cilostamide treatment (**c**, **d**), or released in the cilostamide-free medium for maturation (**e**, **f**). YFP and mCherry signals were recorded by time-lapse microscopy every 15 min for 20 h. The YFP signals were normalized by the level of mCherry signals. The translation rate was calculated for each oocyte by linear regression of the reporter data. Experiments were repeated three times and the data are shown as the mean $\pm$ SEM. One-way ANOVA were used to evaluate statistical significance, ns not significant, **c** **P = 0.0076, ***P = 0.0002, **d** *P = 0.034, **P = 0.0015, **e** *P = 0.025, ***P = 0.0001, **f** **P = 0.0048, ****P < 0.0001. Source data are provided as a Source Data file.

If de-repression of *Ccnb1* translation in GV oocytes were due exclusively to the decreased *Cpeb1* levels detected in old oocytes, increasing the accumulation of CPEB1 protein should rescue this translation phenotype. To test this possibility, GV oocytes were injected with in vitro-translated *Cpeb1* mRNA together with the *Ccnb1* reporter, and the accumulation of YFP was measured. Control experiments where CPEB1 protein levels were measured by western blot indicated an increase in CPEB1 protein levels up to twofold compared to control (Fig. 7a, b). Under these conditions, the increase in accumulation of the *Ccnb1* reporter in old oocytes was reversed by co-injection with the *Cpeb1* mRNA. The rate of translation in these co-injected oocytes was identical to young controls and significantly decreased when compared to old controls (Fig. 7c). Similar rescue of the de-repression was observed when young CPEB1 heterozygous oocytes were injected with reporter and *Cpeb1* mRNA (Fig. 7d).

### The effect of decreased CPEB1 level on translation during oocyte maturation and fertility

**Fig. 8:**
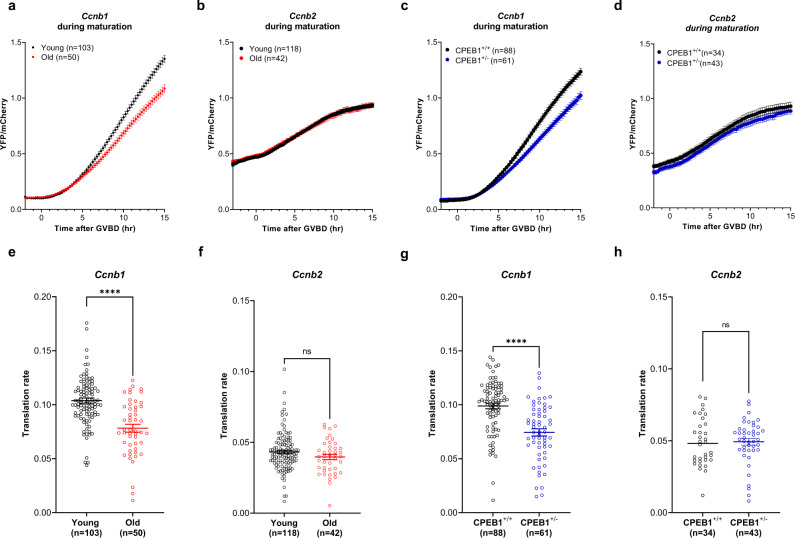
The effect of decreased CPEB1 level on Ccnb1 translation during oocyte maturation. GV oocytes from young and old mice (**a**, **b**, **e**, **f**), or GV oocytes from *Cpeb1*^*+/+*^*;Zp3Cre* mice (CPEB1^+/+^ oocytes) and *Cpeb1*^*fl/+*^*;Zp3Cre* (CPEB1^+/−^ oocytes) (**c**, **d**, **g**, **h**) were injected with 12.5 ng/µl polyadenylated mCherry and 12.5 ng/µl *YFP-Ccnb1* 3’-UTR (**a**, **c**, **e**, **g**) or *YFP-Ccnb2* 3’-UTR (**b**, **d**, **f**, **h**). After pre-incubation overnight, oocytes were released in the cilostamide-free medium for maturation. YFP and mCherry signals were recorded by time-lapse microscopy every 15 min for 20 h. The YFP signals were normalized by the level of mCherry signals. **a**–**d** YFP/mCherry signal in each oocyte was plotted depending on the time of GVBD. **e**–**h** Translation rate was calculated for each oocyte by linear regression of the reporter data between 5 and 10 h (**a**, **c**) or between 3 and 7 h (**b**, **d**). Experiments were repeated three times and the data are shown as the mean $\pm$ SEM. Two-tailed unpaired Student’s *t* tests were used to evaluate statistical significance, ns not significant, ****P < 0.0001. Source data are provided as a Source Data file.

**Fig. 9:**
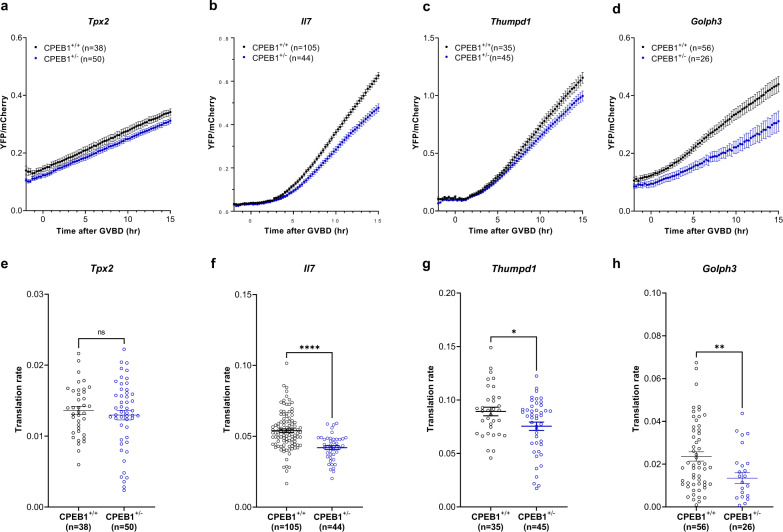
Haploinsufficiency of CPEB1 recapitulates the decreased translation during oocyte maturation. GV oocytes from *Cpeb1*^*+/+*^*;Zp3Cre* mice (CPEB1^+/+^ oocytes) and *Cpeb1*^*fl/+*^*;Zp3Cre* mice (CPEB1^+/−^ oocytes) were injected with polyadenylated mCherry and *YFP-Tpx2* 3’-UTR (**a**, **e**), *YFP-Il7* 3’-UTR (**b**, **f**), *YFP-Thumpd1* 3’-UTR (**c**, **g**), or *YFP-Golph3* 3’-UTR (**d**, **h**). After pre-incubation overnight, oocytes were released in the cilostamide-free medium for maturation. YFP and mCherry signals were recorded by time-lapse microscopy every 15 min for 20 h. The YFP signals were normalized by the level of mCherry signals. **a**–**d** YFP/mCherry signal in each oocyte was plotted depending on the time of GVBD. **e**–**h** Translation rate was calculated for each oocyte by linear regression of the reporter data between 5 and 10 h (**a**, **d**) or between 7 and 12 h after GVBD (**b**, **c**). Experiments were repeated three times and the data are shown as the mean $\pm$ SEM. Two-tailed unpaired Student’s *t* tests were used to evaluate statistical significance, ns not significant, *P = 0.017, **P = 0.0070, ****P < 0.0001. Source data are provided as a Source Data file.

Upon reentry into meiosis, CPEB1 switches its function from repressor to activator of translation. We tested whether translation of *Ccnb1* during maturation is also affected with maternal aging, as we have shown for *Il7, Thumpd1*, and *Golph3* (Fig. 2f–k). The rate of translation of the *Ccnb1* reporter was significantly decreased in old oocytes during maturation (Fig. 8a, e), whereas no differences in *Ccnb2* were observed (Fig. 8b, f). Decreased translation of *Ccnb1* reporter was also detected in young oocytes carrying a CPEB1 haploinsufficiency, with no significant change in *Ccnb2* reporter translation during oocyte maturation (Fig. 8c, d, g, h). In both old oocytes and oocytes with haploinsufficiency, overexpression of *Cpeb1* rescued the decreased *Ccnb1* reporter translation (Fig. 7e, f). Again, there was a *Cpeb1*-gene dosage effect of CPEB1 on the translation of *Ccnb1* reporter (Supplementary Fig. 9b). Translation of *Il7, Thumpd1*, and *Golph3* reporters was also significantly decreased in CPEB1^+/-^ oocytes during maturation (Fig. 9b–d, f–h) while the translation of *Tpx2* was unchanged (Fig. 9a, e). All these results are fully consistent with the result of RNA-IP with CPEB1 antibodies (Supplementary Fig. 8). Thus, dysfunction of CPEB1 during maternal aging affects translation during maturation as well.

**Fig. 10:**
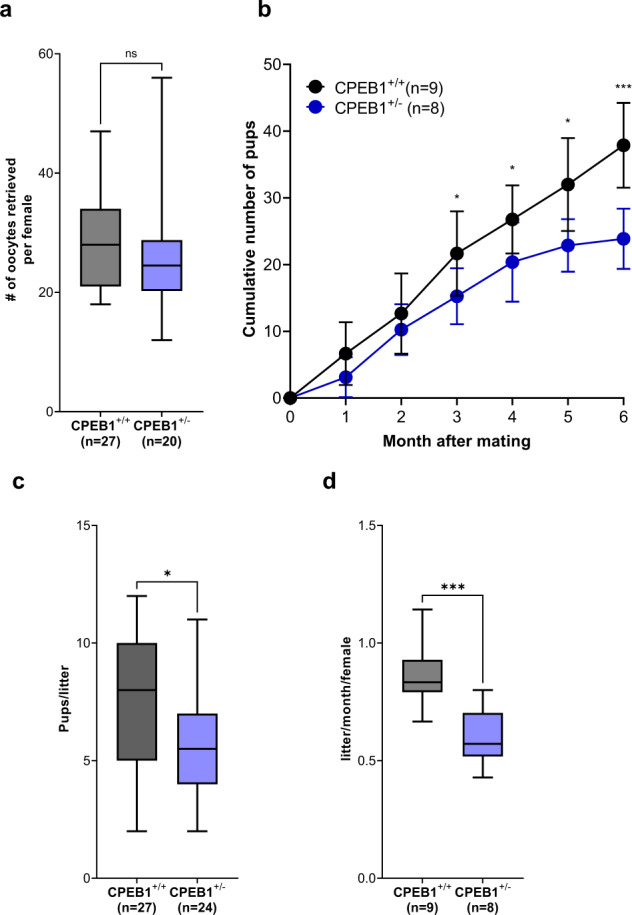
The effect of decreased CPEB1 level on mouse fertility. **a** *Cpeb1*^*+/+*^*;Zp3Cre* mice (CPEB^+/+^ mice) and *Cpeb1*^*fl/+*^*;Zp3Cre* mice (CPEB^+/−^ mice) were primed with PMSG and ovaries were dissected 44 h later. GV oocytes from each pair of ovaries were counted. Data are reported as box and whisker plots. The central line denotes the median value, while the box refers to the 25th to 75th percentiles and whiskers mark minimum and max values. Unpaired Student’s *t* tests were used to evaluate statistical significance, n.s. not significant. **b**–**d** CPEB1^+/+^ and CPEB1^*+/*−^ female mice were mated with WT male mice. Breeding was initiated when mice reached at least 8 weeks of age. **b** Cumulative number of pups per female derived from CPEB1^+/+^ and CPEB1^+/−^ mice was recorded. The data are reported as the mean $\pm$ SD. Two-tailed multiple unpaired Student’s *t* tests were used to evaluate statistical significance at each time point. *P = 0.027 for 3 months, *P = 0.030 for 4 months, *P = 0.0052 for 5 months, ***P = 0.00011 for 6 months. **c** The number of pups per litter up to three pregnancies was recorded and data reported as box and whisker plots. The central line denotes the median value, while the box refers to the 25th to 75th percentiles and whiskers mark minimum and max values. The number of litters analyzed is included among brackets. Two-tailed unpaired Student’s *t* tests were used to evaluate statistical significance, *P = 0.030. **d** the frequency of parturition was measured and expressed as a number of litters per month per female. The data are plotted as box and whisker plots. The central line denotes the median value, while the box contains the 25th to 75th percentiles and whiskers mark minimum and max values. Two-tailed unpaired Student’s *t* tests were used to evaluate statistical significance, ***P = 0.0008. Source data are provided as a Source Data file.

We have previously shown that decreased *Ccnb1* translation causes increased aneuploidy rate and infertility^46^. Given that change in timing of meiosis reentry and decreased translation of *Ccnb1* in oocytes causes infertility, we tested whether a partial loss in CPEB1 expression in oocytes may affect fertility by mating CPEB1^+/−^ female mice with wild-type male mice. Conditional CPEB1 knockout after birth in female mice leads to complete infertility^15,49^. Although there was no difference in the number of oocytes retrieved (Fig. 10a), maturation rate, and diameter of oocytes between CPEB1^+/+^ and CPEB1^+/−^ mice (Supplementary Fig. 10a–c), fertility was significantly decreased 3 months after mating (Fig. 10b) and CPEB1^+/−^ mice progressively produced significantly fewer pups compared with CPEB1^+/+^ mice (Fig. 10c). CPEB1^+/−^ mice also show a decrease in the number of pregnancies (Fig. 10d). The age of first pregnancy of CPEB1^+/+^ and CPEB1^+/−^ mice and the body weight of pups were comparable (Supplementary Fig. 10d, e). Thus, *Cpeb1* haploinsufficiency in young mice causes a decrease in oocyte quality and recapitulates some of the phenotypes associated with CPEB1 dysfunction in maternal aging.

## Discussion

Using different complementary approaches, we have determined that maternal aging is associated with a disruption of the mRNA translation program executed during oocyte maturation. Approximately 5% of the mRNAs present in the oocyte are incorrectly translated during maturation. Although changes in translation are not profound, we propose that the cumulative effects of inefficient translation and consequently altered proteostasis have an impact on oocyte quality. Mechanistically, we document that translation of the mRNA coding for an RBP critical for translation regulation in the oocyte, CPEB1, is affected, resulting in decreased accumulation of this protein. This, in turn, results in altered translation in oocytes and aberrant progression through the meiotic cell cycle. The association of CPEB1 with these phenotypes detected in old oocytes is recapitulated by a model of haploinsufficiency of CPEB1 where similar reductions in CPEB1 protein are induced in young oocytes. Moreover, increasing CPEB1 levels in old oocytes rescues these translation phenotypes. Therefore, we believe that altered CPEB1 function, likely arising during oocyte growth, is the molecular defect underlying some of the phenotypes associated with decreased oocyte fitness during maternal aging. Together with decreased production of oocytes due to follicle depletion, the altered CPEB1-dependent disruption of de novo protein synthesis may recapitulate the decreased fertility observed with aging.

Our RiboTag IP/RNA-Seq data show an alteration in ribosome loading in old oocytes when compared to young oocytes. Although the power of this analysis was limited by the availability of old oocytes for molecular analysis, our data are internally consistent. For instance, the TE in control young oocytes of this study is highly correlated to that measured in other datasets we have generated^14,15^, as well as the RNA-Seq data on translation reported by others^50^. Comparison between our data and the translatome of Pan et al^24^. shows consistent effects on some genes. Moreover, GO analysis predicts a highly significant disruption in the translation of components necessary for mitochondrion biogenesis/function. Defective mitochondrion function is a hallmarck of aging^37^, and altered translation of mitochondrial proteins have been reported. The prediction of an altered ribosome-associated protein acetylation and ribosome biogenesis also is supported by our finding of altered polyadenylation of *Thumpd1* mRNA and altered translation of a reporter driven by the 3’-UTR of *Thumpd1*. However, comparison of our data with those recently reported by others^27^, unfortunatelly shows little or no overlap, with the possible exception of a decrease in *Sgk1* translation. Although that report documented altered translation of 38 mRNAs (FDR < 0.1; 8 mRNAs with FDR < 0.05), we detect more widespread defects that amount to 726 mRNAs if the same cutoff is applied (FDR < 0.1, 256 mRNAs with FDR < 0.05). The reason for this discrepancy is at presently not clear. It shoud be noted that Del Llano et al.^27^ assessed translation immediately after in vitro GVBD, a time during which activation/repression of translation is minimally different from that in quiescent oocytes^15^. Conversely, we assessed changes in translation in MII oocytes matured in vivo, when differences in translation have reached a maximum.

Collectively, our data point to a defective function of CPEB1 as one of the molecular causes of altered translation. This conclusion is based on (1) Altered translation of the *Cpeb1* mRNA in oocytes and related 50% decrease in CPEB1 protein; (2) Defective translation of *Ccnb1*, *Il7 Thumpd1*, and *Golph3* mRNAs, which are all bona fide CPEB1 targets; (3) Altered polyadenylation of *Ccnb1*, *Il7 Thumpd1*, and *Golph3*; (4) Altered parameters of oocyte reentry into the cell cycle predicted by altered regulation of CCNB1 synthesis; (5) A defective accumulation of CPEB1 in young oocytes completely recapitulates the translation and meiotic progression observed in old oocytes, and increasing CPEB1 protein levels in old oocytes rescues the translation phenotypes. Therefore, our conclusions are based on multiple independent observations all converging on CPEB1 dysfunction during aging. It should be noted that, although not detected by some^51^, others have reported that maternal aging causes an accelerated reentry into the meiotic cell cycle^52,53^, as we have detailed here. Moreover, although the detected effects associated with *Cpeb1* haploinsufficiency are not major, we consistently observe a *Cpeb1*-gene dosage-dependent effect, reinforcing the validity of our observations.

Bioinformatic analysis of the properties of the transcripts affected during aging indicates that not all associated 3’-UTRs contain a putative CPE. Of the 126 3’-UTRs analyzed, we can identify a putative CPE in 84, whereas this cis-acting element is not detected in 42 3’-UTRs. This is documented by the analysis of *Nlrp5* mRNA. The translation of the endogenous *Nlrp5* mRNA is increased in our dataset, and an apparent transcript increase is detected in the deposited aging transcriptome. Moreover, reporter data show defective repression of *Nlrp5* translation. The *Nlrp5* 3’-UTR does not contain discernible CPE elements, and the endogenous *Nlrp5* is not immunoprecipitated in a RIP assay; thus, mechanisms other than CPEB1-mediated translation may be altered. That not all affected transcripts contain a CPE is also inferred with the analysis of the mRNAs coding for the mitochondrial ribosomal proteins. These findings suggest that either translation of some mRNA is indirectly affected by a dysfunctional CPEB1 or that more complex events disrupt the translation of maternal mRNAs during aging. In this latter scenario, a CPEB1-dependent component acts in parallel with an alteration of other regulatory circuits controlling translation. The RBPs involved, as well as the exact molecular mechanisms implicated, require further investigation.

Given the increased translation in GV oocytes followed by a decreased rate of translation during maturation, we calculate that the overall activation of *Ccnb1* mRNA translation is decreased by 50% in old oocytes. This decrease is substantial and likely disruptive of meiotic progression. For instance, we find that a 30% decrease in *Ccnb1* mRNA translation in *Ccnb2*-loss of function is associated with a significant increase in aneuploidy^46^. The kinetochore-microtubule attachment is regulated by CDK1 activity and a change in CDK1 activity leads to lagging chromosomes at anaphase I^54^. In addition, studies have reported that pharmacological acceleration of meiosis I cause higher rate of aneuploidy^55,56^. Thus, change in timing of meiosis reentry and altered CDK1 activation may be linked to the aneuploidy described in aged oocytes.

The post-transcriptional regulation controlling *Cpeb1* mRNA translation has not been investigated extensively. However, CPEB1 binds to its own mRNA (Supplementary Fig. 8) and represses its own translation in GV oocytes^57^. Moreover, an element that binds AU-rich binding factor 1 (AUF1) has been described, and a function for microRNA-dependent modulation of *Cpeb1* has been described in neuroblastoma cells^58^. Thus, several possible mechanisms may be causing a decreased translation of *Cpeb1* during oocyte growth and maturation, mechanisms that require further investigation.

Collectively, our data strongly support the concept that a disruption in the translation program in mouse oocytes is associated with maternal aging. Moreover, we provide evidence that an altered stoichiometry of CPEB1 protein may be one of the molecular defects causing altered maternal mRNA translation in aging oocytes. In addition to being consistent with other observations, the fact that these phenotypes can be rescued by overexpression of CPEB1 and recapitulated by a model of *Cpeb1* haploinsufficiency in oocytes from young females provides compelling support of our overall hypothesis. It should be also noted that heterozygous deletions of the *Cpeb1* gene in humans have been associated with Premature ovarian insufficiency^59–62^, a view that further supports the *Cpeb1*-dependent phenotypes during aging. A disrupted CPEB1-dependent mode of the translation may not be the only mechanism, as preliminary evidence also suggests disruption of a CPEB1-independent component of translation. Further studies are required to determine how all these defects in the translation program cumulatively converge to disrupt oocyte quality and fertility.

It should be pointed out that altered translation is just one of the components associated with reproductive aging. Although not profound, the decreased oocyte quality and fertility in young mice associated with *Cpeb1* haploinsufficiency on a background of normal number of ovulated oocytes, likely compounds with the large decrease in the number of follicles that mature and the number of ovulated oocytes during aging. Thus, we propose that the overall decrease in fertility observed during aging is the sum or the compound effects of the altered number of oocytes produced and their defective developmental competence due to altered maternal mRNA translation.

## Methods

### Experimental animals

All experimental procedures involving animal models used were approved by the Institutional Animal Care and Use Committee of the University of California at San Francisco (AN182026-03A). All animals used were of the C57BL/6 inbred strain. Mice were housed in 12 h light/12 h dark cycle at the temperature of 18−23 °C with 40−60% humidity environment. Young mice used were 1-month-old females, whereas 12–15 months old retired female breeders were considered old mice. C57BL/6-Zp3CreRpl22tm1.1Psam (*RiboTag*^*F/F*^*;Zp3-Cre*) mice were obtained from Jackson Laboratories and bred as described previously^29^. CPEB1-targeted mice were a gift from ref. ^63^ and C57BL/6-*Cpeb1*^*F/+*^;*Zp3-Cre* female mice were bred in our facility.

### Oocyte collection

Female mice were injected with 5 I.U. pregnant mare serum gonadotropin (PMSG; MyBioSource) to induce follicle growth and sacrificed 44 h after injection. The ovaries were dissected, and COCs were collected in media containing HEPES-modified minimum essential medium Eagle (Sigma-Aldrich, M2645) supplemented with 0.23 mM sodium pyruvate (Gibco, 11360070), penicillin–streptomycin (Genesee Scientific Corporation (GenClone), 25-512), and 3 mg/ml BSA (Sigma-Aldrich, SIAL-A3311), 6 mM sodium bicarbonate (JT-Baker, 3506-1), and a PDE inhibitor (1 µM cilostamide (Calbiochem, 231085) or 2 μM milrinone (Enzo ALK-270-083)). Aspiration through a glass pipette allowed for the removal of the surrounding cumulus cells. Denuded oocytes were maintained at the GV stage in α-MEM (Gibco, 12561-056) supplemented with 0.23 mM sodium pyruvate, penicillin–streptomycin, and 1 µM cilostamide (or 2 μM milrinone) at 37 °C under 5% CO_2_. For the collection of in vivo MII oocytes, female mice were injected with 5 I.U. of PMSG followed by 10 I.U. human chorionic gonadotropin (hCG; Goldline Labs) 48 h after PMSG injection, and after 13 h, sacrificed for ovulated oocytes retrieval from the fallopian tubes.

### RiboTag IP

Oocytes from *RiboTag*^*F/F*^*;Zp3-Cre* female mice stimulated with 5 I.U. PMSG followed by 10 I.U. hCG, or ovaries from *RiboTag*^*F/F*^*;Zp3-Cre* female mice stimulated with 5 I.U. PMSG only was used for RiboTag-immunoprecipitation. Oocytes were collected in 10 µl of 1× PBS (Invitrogen, AM9625), supplemented with 0.1% w/v polyvinylpyrrolidone (PVP; Sigma, P0930), 0.1% v/v Cyclohexamide (CHX; Sigma C7698), and 250 U RNAseOUT (Invitrogen 10777-019), then flash-frozen in liquid nitrogen, and stored at −80 °C. Ovaries were collected whole, flash-frozen in liquid nitrogen, and stored at −80 °C. To prepare samples for the IP, the appropriate volume (80 µl per sample) of Dynabeads™ Protein G (Invitrogen, 10004D) was washed two times in 250 µl homogenization buffer (HB: 50 mM Tris–HCl pH 7.4, 100 mM KCl, 12 mM MgCl_2_ and 1% v/v NP-40) on a rotor at 4 °C for 5 min per wash. Two additional washes were performed with 250 µl supplemented HB (sHB: HB supplemented with 1 mM DTT (sigma 646563), 1× HALT protease inhibitor (Fisher P178441), 200 units/ml RNaseOUT, 200 units/ml RNasin (Promega N2115), 100 µg/ml CHX) on a rotor at 4 °C for 5 min per wash. Following the final wash, the beads were eluted back to the original volume with sHB. Oocyte samples were thawed and pooled to yield a total of 200 oocytes per replicate, and 300 µl sHB was added to each pooled sample. To lyse the cells, samples were vortexed for 30 s and the homogenates were centrifuged for 15 min at 20,817×*g* at 4 °C. Ovaries were homogenized in 300 µl sHB and centrifuged likewise. To pre-clear the samples, the supernatants were transferred to new tubes, 20 µl of washed beads were added to each supernatant, and samples were incubated on a rotor at 4 °C for 30 min. A magnetic rack was used to remove the beads, and 30 µl (10%) of each pre-cleared lysate was collected and added to 250 µl RLT buffer (Qiagen, 74034) supplemented with 1 mM DTT to serve as the input samples. The input samples were frozen and kept at −80 °C until RNA extraction. In total, 4 µl (4 µg) anti-HA.11 epitope tag antibody (1/75, Invitrogen, 26183) was added to each of the extracts and all samples were incubated on a rotor at 4 °C for 4 h. Thirty microliters of washed beads were added to the samples and incubated overnight on a rotor at 4 °C. The beads (now bound by HA-tagged ribosomes and the associated mRNAs) were washed four times in 750 µl urea wash buffer (uWB: 50 mM Tris–HCl pH 7.4, 150 mM KCl, 12 mM MgCl_2_, 1% v/v NP-40, 1× protease inhibitors, 1 mM DTT, 40 U RNaseOUT, 40 U RNasin, 100 µg/ml cycloheximide, and 1 M urea) on a rotor at 4 °C for 10 min per wash. The bead slurry was then transferred to fresh tubes, pelleted via magnetic rack, and the uWB removed. To strip the mRNA complex off the beads, 250 µl RLT buffer supplemented with DTT was added to each sample and samples were vortexed for 30 s. Finally, Beads were pelleted and removed before RNA extraction was performed using the Rneasy Plus Micro Kit (Qiagen, 74034). Samples were eluted in 14 µl of RNase-free water and used downstream for RNA-Seq or RT-qPCR analysis.

### RNA-Seq

#### RNA-Seq library preparation and sequencing

RiboTag RNA samples were sent to the Gladstone Institutes Genomics Core for quality control using Bioanalyzer (Agilent) and cDNA library preparation. cDNA was prepared with the Ovation RNA-Seq System V2 Kit (NuGEN, 7102), which uses a proprietary combination of enzymes and primers to preferentially prime non-rRNA sequences. First-strand cDNA synthesis is achieved with random hexamer primers and chimeric poly(T) primers, which bind to the region spanning the end of the 3’-UTR and the start of the poly(A) tail. Because stable mRNAs have at least 20 adenosine residues, the use of these chimeric poly(T) primers allows for preferential priming of adenylated messages (mRNAs) with minimal bias for messages with longer poly(A) tails. RNA-Seq libraries were constructed using the Ovation Ultralow System V2 (NuGEN, 0344) and analyzed by Bioanalyzer, quantified by qPCR (Kapa Biosystems, KR0405), and sequenced using the HiSeq 4000 system (Illumina).

#### Sequence quality assessment and trimming

The quality of the raw sequence data was checked via FASTQC. The sequence files were then trimmed using tools in Trimmomatic 0.36^64^. The following were removed: Illumina TruSeq3 single-ended adapter sequences, bases with a quality score <3 at the start and end of a read, bases that had an average quality per base of <15 calculated using a sliding window to average four bases, and any reads that were shorter than 36 bases. The reads were single-ended and qualities were ASCII characters equal to the Phred quality plus 33.

#### Mapping and counting reads

HiSat2^65^ was used to build indexes from the Reference Consortium Mouse Build 38 (mm10) and to align sequence reads to the genome. The resulting .bam files were sorted and indexed with SAMtools^66^. Count files for each group were created with HTSeq using the mouse GENCODE Gene set release M11. The input data were.bam files, the data were not from a strand-specific assay, and the feature type used was “gene”.

#### Differential expression (DE) analysis

The Bioconductor packages edgeR^67^ and limma^68^ were used for statistical analyses. Only reads with greater or equal to 10 counts per million reads (CPM) in at least two samples were kept. Trimmed mean of *M*-values (TMM) normalization, which accounts for compositional differences among the libraries, was then performed on HA reads and input reads, separately. Using the raw counts, dispersion, and design matrix, the negative binomial generalized linear model was fitted for each gene. Finally, pairwise likelihood ratio tests for young versus old was conducted. Translational efficiency was calculated by using the ratio of the amount of mRNA recovered in the RiboTag pellet (HA-IP) over the total mRNA present in the oocyte (Input).

#### Gene ontology (GO) analysis

Gene lists were uploaded to DAVID 6.8^69,70^ and processed with the Functional Annotation Tool.

### RNA-IP

The appropriate volume (80 µl per sample) of Dynabeads™ Protein G was washed twice in 250 µl incomplete lysis buffer (iLB: 30 mM Tris–HCl pH 7.4, 150 mM KCl, 10 mM MgCl_2_, 0.5% v/v NP-40, 0.25 mM Na_3_VO_4_ and 10 mM β-glycerophosphate) on a rotor at 4 °C for 5 min per wash. Two additional washes were performed with 250 µl complete LB (cLB: iLB supplemented with 5× HALT protease inhibitor, 1 mM DTT, 80 U/ml RNAseOUT, 80 U/ml RNasin and 2× ribonucleoside vanadyl complex) on a rotor at 4 °C for 5 min per wash. The final wash solution was removed, and the beads were eluted to the original volume with cLB. Two hundred wild-type GV oocytes were collected in 10 µl 0.1% w/v PVP and 250 U RNAseOUT in 1× PBS, flash-frozen in liquid nitrogen, and stored at −80 °C. To homogenize the cells, 300 µl of cLB was added, samples were vortexed for 30 s, and then incubated on ice for 10 min. The homogenates were then centrifuged for 15 min 20,817×*g* at 4 °C, and the supernatants were transferred to new tubes. In total, 30 µl (10%) of each supernatant was saved in 250 µl RLT buffer supplemented with 1 mM DTT as input samples and the remaining volume was equally divided into the CPEB1-IP and the IgG-IP (control). The volume of each sample was increased to 300 µl with cLB, 4 µl (4 µg) of the appropriate antibody (1/75, anti-CPEB1: Abcam, ab73287 and control IgG: Millipore, 12-371) was added to each tube, and samples were incubated on a rotor for 4 h at 4 °C. Thirty microliters of washed beads were then added to each tube and samples were incubated on a rotor overnight at 4 °C. The beads of each sample were pelleted using a magnetic rack and washed 4 times on a rotor at 4 °C with 750 μl wash buffer (WB: 30 mM Tris–HCl pH 7.4, 200 mM KCl, 10 mM MgCl_2_, 0.5% v/v NP-40, 0.25 mM Na_3_VO_4_, 10 mM β-glycerophosphate, 1× HALT protease inhibitor, 1 mM DTT, 1 M urea, 50 U/ml RNaseOUT, and 50 U/ml RNasin) for 10 min per wash. After each wash, the beads were pelleted and the WB removed. Samples were transferred to a clean tube, and 250 µl RLT buffer supplemented with 1 mM DTT was added to each sample. Samples were vortexed for 30 s, the beads were pelleted and removed. RNA extraction was performed following the Qiagen Rneasy Plus Micro Kit protocol. Samples were eluted in 14 µl of RNase-free water and used for downstream RT-qPCR analysis.

### RNA extraction and real-time quantitative PCR (RT-PCR)

Extracted RNA was reverse-transcribed using the SuperScript^TM^ III First-Strand Synthesis System with random hexamer or oligo dT primers (Invitrogen, 18080-051). Gene expression was measured using TaqMan^TM^ Assays and Taqman^TM^ Fast Advanced Master Mix (ThermoFisher, 4444557). TaqMan Assays used were as follows; *ActB* (Mm02619580_g1), *Ccnb1* (Mm03053893_gH), *Ccnb2* (Mm01171453_m1), *Cpeb1* (Mm01314928_m1), *Dppa3* (Mm01184198_g1), *Eif4a1* (Mm03807704_g1), *Gata4* (Mm00484689_m1), *Golph3* (Mm00499420_m1), *Il7* (Mm01295803_m1), *Nlrp5* (Mm01143609_m1), *Thumpd1* (Mm00524010_m1), *Tpx2* (Mm01245970_m1), *Sirt1* (Mm01168521_m1), *Sirt7* (Mm01248607_m1), *Rpl19* (Mm02601633_g1), and *Zp3* (Mm00442176_m1). Preliminary qPCRs were done to test mRNA levels not changed in GV and MII oocytes. Among the tested genes, *Actb* and *Eif4a1* were expressed at similar levels in GV and MII when random hexamers were used for RT priming, while *Dppa3* and *Rpl19* were stable when oligo(-dT) were used. Since no genes were found to be stable with both priming conditions, we used the geometric mean of the four best candidates for the two priming conditions, and no differences were observed in the Ct values for either the meiotic stage or the priming method (*N* = 6; paired, one-way ANOVA). Therefore, in all the experiments using qPCR the geometric mean of *Actb*, *Eif4a1, Dppa3*, and *Rpl19 Ct* was used for data normalization. Ten microliter reactions were run on the QuantStudio 6 Flex Real-time PCR System (Applied Biosystems). Gene expression was quantified via the 2^−Ct^ method, and statistical analysis was performed via GraphPad Prism 8.

### Reporter mRNA preparation

The *Ccnb1*, *Ccnb2*, *Golph3*, *Il7*, *Nlrp5*, *Thumpd1*, and *Tpx2* 3’-UTR and *Cpeb1* open reading frame sequences (Supplementary Data 3) were obtained by amplification of mRNA from oocytes with appropriate primers (Supplementary Data 4). YFP reporters, mCherry, and FL reporters were subcloned in the pcDNA 3.1 vector and RL reporters were prepared in the pRL-TK vector, both containing a T7 promoter that allows in vitro transcription to synthesize mRNAs, and fidelity was confirmed by DNA sequencing. All reporters included a stretch of 20A at the 3’ end. The mCherry reporter was in vitro polyadenylated and contained a poly(A) tail of 150–200 A. mRNA reporters were transcribed in vitro to synthesize mRNAs with mMESSAGE mMACHINE T7 Transcription Kit (Ambion, AM1344); polyadenylation of mCherry and Firefly luciferase was obtained using Poly(A) Tailing Kit (Ambion, AM1350). All the messages were purified using MEGAclear Kit (Ambion, AM1908). mRNA concentrations were measured by NanoDrop, and its integrity was evaluated by electrophoresis.

### Luciferase assay

Oocytes still enclosed in the cumulus cells (CEOs) were injected with 12.5 ng/μl *Il7-RL*, *Thumpd1-RL*, or *Tpx2-RL* and 12.5 ng/µl of polyadenylated FL using a FemtoJet Express programmable microinjector with an Automated Upright Microscope System (Leica, DM4000B). Injected CEOs were pre-incubated for 3 h in culture medium supplemented with 2 μM milrinone, and then cultured in milrinone-free medium supplemented with 100 nM amphiregulin. After 16 h, the CEOs were denuded, collected in lysis buffer and frozen. Luciferase activities in the oocyte extracts were measured using a dual-luciferase reporter assay kit (Promega), and luminescence was detected with a SpectraMax L luminometer (Molecular Devices). Data are reported as ratios of RL and FL.

### YFP reporter assay

*Ccnb1-YFP*, *Ccnb2-YFP*, *Il7-YFP*, *Golph3-YFP*, *Thumpd1-YFP, or Tpx2-YFP* were injected in oocytes at 12.5 ng/µl with polyadenylated *mCherry* at 12.5 µg/µl using a FemtoJet Express programmable microinjector with an Automated Upright Microscope System. After pre-incubating 3 h or overnight in α-MEM medium with 1 µM cilostamide, oocytes were released in cilostamide-free medium for in vitro maturation or incubated with cilostamide to maintain GV state. Time-lapse recordings were performed using a Nikon Eclipse T2000-E equipped with a mobile stage and environmental chamber of 37 °C and 5% CO_2_ at the following settings: filter set, dichroic mirror YFP/CFP/mCherry 69008BS; Ypet channel (Ex: S500/20×49057 Em: D535/30 m 47281); and mCherry channel (Ex: 580/25×49829 Em: 632/60 m). Images were processed and fluorescence was quantified using MetaMorph (Molecular Devices). Ratios of YFP reporter of the plateaued level of mCherry signal were reported as the protein accumulation during oocyte maturation. The rate of translation were calculated with YFP/mCherry ratios as the slope of the simple linear regression curve at the indicated time interval.

### In vitro CDK1 kinase assay

Oocytes were collected in 30 µl of 2× kinase buffer (100 mM HEPES, 30 mM MgCl_2_, 2 mM EGTA, 10 mM CaCl_2_, 2 mM DTT, 2 µg/ml leupeptin, 2 µg/ml aprotinin, 2 µM okadaic acid). Oocytes were lysed by freezing and thawing twice in liquid nitrogen. Extracts were incubated at 30 °C for 30 min in the presence of 0.1 mM ATP, 10 mM DTT, 2 µM okadaic acid, and 2 µg of recombinant peptide GST-PP1 as the substrate. PP1-GST was produced as previously described^47^. Reactions were stopped by adding Laemmli sample buffer and boiling at 95 °C for 5 min. CDK1 activity was measured by quantifying the western blot signal of phosphorylated T320 of the total substrate loaded evaluated by Ponceau S staining.

### Western blotting

Oocytes were lysed in 10 µl of 0.1% w/v PBS/PVP with 4 μl Laemmli buffer (Bio-Rad, 161-0747) with β-mercaptoethanol (Gibco 21985023), a phosphatase inhibitor, and protease inhibitor. The oocyte lysates were boiled for 5 min at 95 °C and left on ice, then separated on 10% v/v or 12% v/v polyacrylamide gels and transferred to a Polyvinylidene difluoride (PVDF) membrane (Millipore ISEQ00010). Membranes were blocked in 5% w/v milk for 1 h at room temperature and incubated with primary antibody overnight at 4 °C. The primary antibody and dilutions used were as follows: CPEB1 (Abcam, ab73287, 1:1000), α-tubulin (Sigma-Aldrich, T6074, 1:10,000), T320-pp1 (Abcam, ab62334, 1:30,000). Membranes were washed in TBS-Tween 20 (0.05% v/v) and incubated with HRP-conjugated secondary antibodies, 1:10,000 anti-rabbit IgG (GE Healthcare, NA934V) and 1:10,000 anti-mouse IgG (GE Healthcare NA931V), for 2 h at room temperature. Signals were detected using Clarity Western ECL substrate (Bio-Rad, 170-5061). The images are analyzed with the ImageJ 1.53a.

### Statistical analysis

Statistical analysis was performed using the GraphPad Prism 9.3.1 package. The statistical analysis performed depended on a specific experiment and is described within the figure legend. For comparison between two groups, a two-tailed paired or unpaired *t* test was used, for multiple comparison, one-way ANOVA was used. Statistical significance is denoted by an asterisk in each graph. The quality of RNA-Seq reads were performed using FastQC and reads were then trimmed with Trimmomatic 0.36. Alignment of the reads to the mouse genome was performed by Hisat2, .bam files were sorted and indexed using Samtools, and count files were generated by HTSeq. TMM normalization and the remaining RNA-Seq statistical analyses were done through edgeR.

### Reporting summary

Further information on research design is available in the Nature Portfolio Reporting Summary linked to this article.

## Supplementary information

Supplementary information

Description of Additional Supplementary Files

Supplementary data 1-4

Reporting Summary

## Data Availability

RiboTag-IP/RNA-Seq data have been deposited in the NCBI Gene Expression Omnibus (GEO) database under the accession code GSE222638. The published available datasets used in this work were from NCBI Gene Expression Omnibus (GEO) accession number GDS3295 [https://www.ncbi.nlm.nih.gov/geo/query/acc.cgi?acc=GSE11667] (Aging transcriptome^24^), GSE35106 (Polysome array^14^), GSE135525 (RiboTag IP/RNA-Seq^15^), GSE165782 (PAIso-seq^71^), and GRCm38 [https://www.ncbi.nlm.nih.gov/assembly/GCF_000001635.20] (mouse genome dataset). The data supporting the findings of this study are available from the corresponding authors upon reasonable request.  Source data are provided with this paper.

## Source data

Source Data
