# Author Correction: CPEB1-dependent disruption of the mRNA translation program in oocytes during maternal aging

**DOI:** 10.1038/s41467-023-36396-1

**Published:** 2023-02-06

**Authors:** Nozomi Takahashi, Federica Franciosi, Enrico Maria Daldello, Xuan G. Luong, Peter Althoff, Xiaotian Wang, Marco Conti

**Affiliations:** 1 Center for Reproductive Sciences, University of California, San Francisco, CA 94143 USA; 2 USA Eli and Edythe Broad Center of Regeneration Medicine and Stem Cell Research, University of California, San Francisco, CA 94143 USA; 3 Department of Obstetrics and Gynecology and Reproductive Sciences, University of California, San Francisco, CA 94143 USA; 4 Present Address: Reproductive and Developmental Biology Lab, Department of Veterinary Medicine and Animal Science, Università degli Studi di Milano, 20133 Milan, Italy; 5 Present Address: Sorbonne Université, CNRS, Laboratoire de Biologie du Développement—Institut de Biologie Paris Seine, LBD-IBPS, Paris, France

**Keywords:** Translation, Ageing

Correction to: *Nature Communications* 10.1038/s41467-023-35994-3, published online 26 January 2023

The original version of this Article contained an error in the last sentence of the Acknowledgements section, which should have read: ‘The authors acknowledge the support of the UCSF Bakar Aging Research Institute for the purchase of aged mice, and Drs. Leanne Jones and Saul Villeda for the helpful discussion.’

This has been corrected in both the PDF and HTML versions of the Article.

