# Supplementary material for: CPEB1-dependent disruption of the mRNA translation program in oocytes during maternal aging
Source: Nat Commun. 2023 Jan 26;14:416. doi: 10.1038/s41467-023-35994-3 (PMC9877008; doi:10.1038/s41467-023-35994-3)
Supplement: Supplementary file 1 — Supplementary information [file 41467_2023_35994_MOESM1_ESM.pdf]

Supplementary information

CPEB1-dependent disruption of the mRNA translation program in oocytes during maternal aging

Nozomi Takahashi et al.

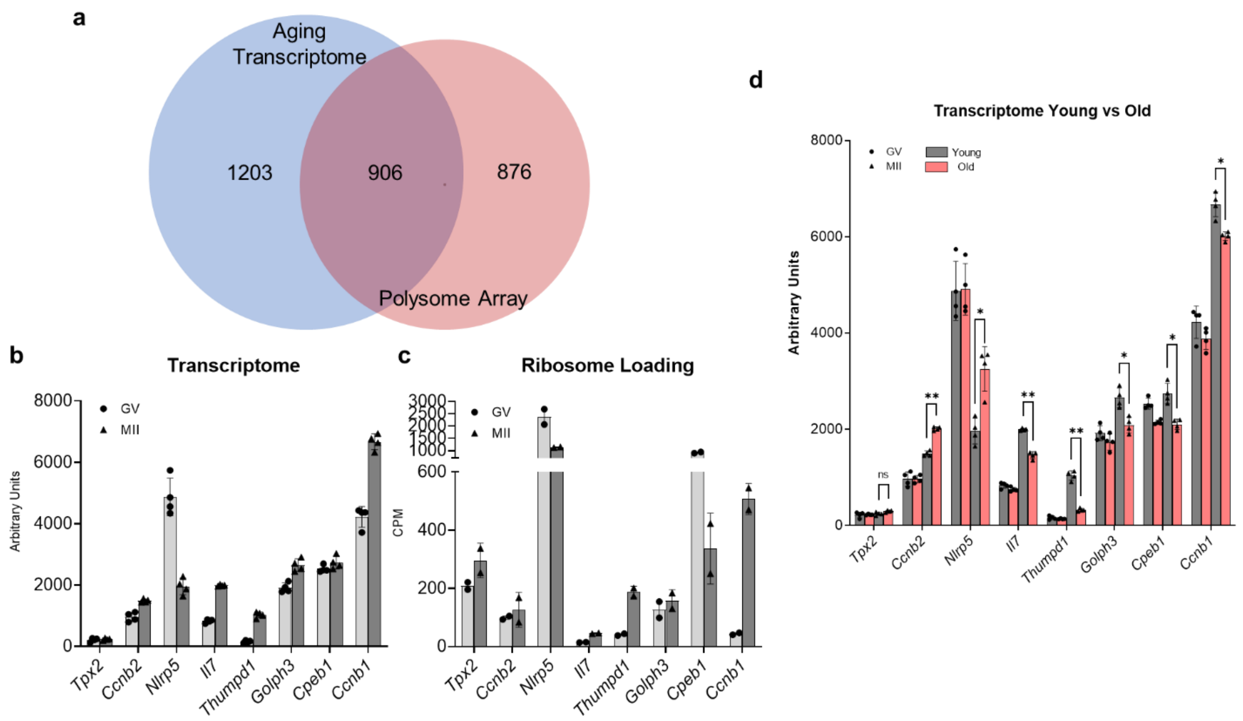

Supplementary Figure 1. Analysis of datasets comparing transcriptome in young and old oocytes and dataset monitoring translation of maternal mRNA

(a) Venn diagram comparing transcripts apparently increasing from germinal vesicle (GV) to MII oocytes in the aging dataset (Accession: GDS3295; ID:3295 [1]) and transcripts recruited to the polysomes in young oocytes (Accession: GSE35106; ID:200035106 [2]). **(b)** Transcripts levels of young oocytes during maturation from the aging dataset [1]. The four biological independent samples are plotted. Data are the mean ± SD. **(c)** Change in ribosome loading mRNA in young oocytes during maturation. Data are the mean ± range of duplicate determination. Data are from deposited RiboTag dataset (Accession: GSE135525 [3]). **(d)** Change in transcripts levels in young and old oocytes from the aging dataset [1]. The four biological independent samples are used. Mean ± SD is shown. Two-tailed multiple paired Student's t-tests were used to evaluate the statistical significance between young and old MII oocytes, ns not significant, ** p=0.0012 for *Ccnb2*, * p=0.032 for *Nlrp5*, ** p=0.0012 for *Il7*, ** p=0.00064 for *Thumpd1*, * p=0.032 for *Golph3*, * p=0.0098 for *Cpeb1*, * p=0.018 for *Ccnb1*. Source data are provided as a Source Data file.

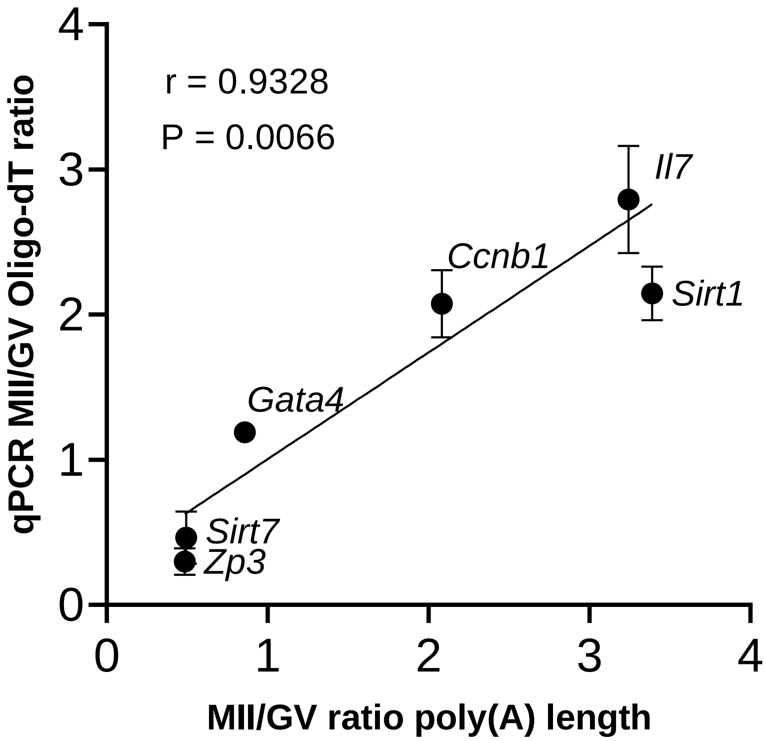

Supplementary Figure 2. Correlation between qPCR oligo(-dT)/random priming ratio and poly(A) tail length

RNA of GV or MII oocytes was reverse transcribed by oligo(-dT) primers, and quantitative PCR was performed with specific primers. Data are normalized by the average expression of *Dppa3*, *Rpl19*, *ActB*, and *Eif4a1*. The $\log_2$ fold changes for GV vs MII oocytes is plotted on the y-axis and the $\log_2$ fold changes for GV vs MII oocytes in poly(A) tail length is shown on the x-axis. n=8 for *Ccnb1,* 1 for *Gata4*, 9 for *Il7*, 4 for *Sirt1*, 2 for *Sirt7*, 3 for *Zp3* biologically independent samples are used. The data are shown as the mean ± SEM. Linear regression curve is shown. Pearson r and p value are included in the panel. The poly(A) data are from PAIso-seq dataset (Accession: GSE165782 [4])**.** Source data are provided as a Source Data file.

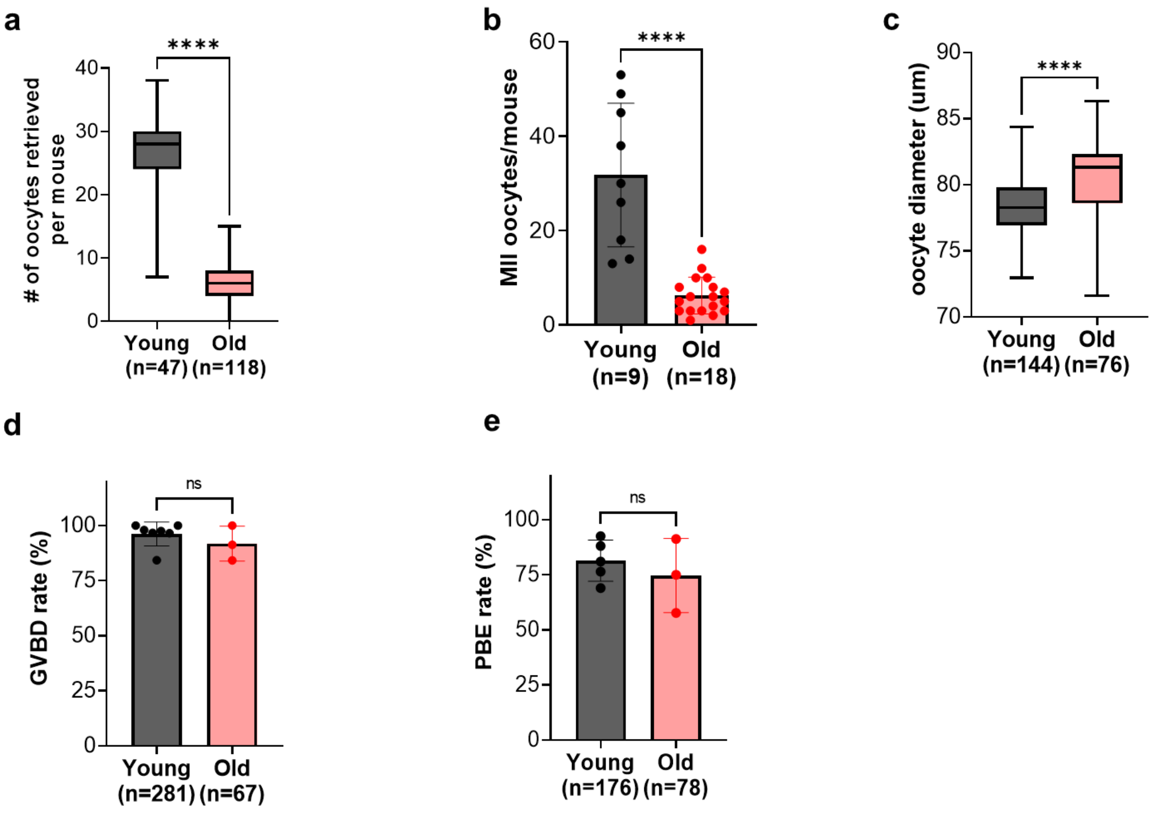

Supplementary Figure 3. Properties of young and old oocytes used in the aging study

(a) Young and old mice were primed with pregnant mare serum gonadotropin (PMSG) and ovaries were dissected 44 hours later. GV oocytes from each pair of ovaries were counted. Box and whisker plots are shown. The central line denotes the median value, while the box refers to the 25th to 75th percentiles and whiskers mark minimum and max values. The number of mice analyzed is reported among brackets. Two-tailed unpaired Student's t-tests were used to evaluate statistical significance, **** $p<0.0001$. **(b)** Young and old mice were primed with PMSG followed by human chorionic gonadotropin (hCG). MII oocytes collected from ampulla were counted. Mean $\pm$ SD is reported. The number of mice analyzed is included among brackets. Two-tailed unpaired Student's t-tests were used to evaluate statistical significance, **** $p<0.0001$. **(c)** Oocyte diameter was measured by inspecting brightfield images. Data are reported as box and whisker plots. The central line denotes the median value, while the box refers to the 25th to 75th percentiles and whiskers mark minimum and max values. The number of oocytes analyzed is included among brackets. Two-tailed unpaired Student's t-tests were used to evaluate statistical significance, **** $p<0.0001$. **(d, e)** GV oocytes from young and old mice were incubated in cilostamide-free medium and brightfield images were captured every 15 min for 20 hours. Germinal vesicle breakdown (GVBD) rates up to 2 hours (d) and polar body extrusion (PBE) rates (e) scored in each experiment. Mean $\pm$ SD is shown. The number of oocytes analyzed is reported among brackets. Two-tailed unpaired Student's t-tests were used to evaluate statistical significance, n.s. not significant. Source data are provided as a Source Data file.

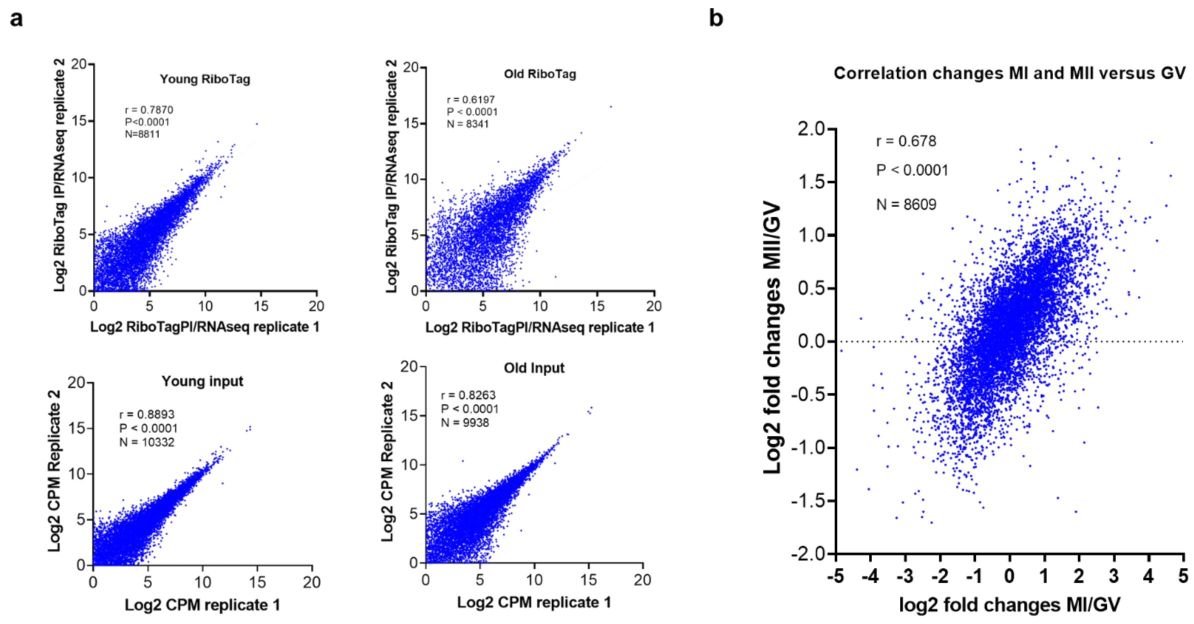

Supplementary Figure 4. Data quality analysis of RiboTag IP/RNA-Seq

(a) Correlation between replicate measurements for the young and old input RNA-Seq and RiboTag IP/RNA-Seq. Two-tailed, non-parametric Spearman correlation was performed comparing the two replicates. Spearman r, p value and number of genes are included in each panel. An increased noise was detected in the old RiboTag IP data set. **(b)** Correlation between the changes in RiboTag IP/RNA-Seq observed for the MII dataset used in this study and our previously published dataset for changes in GV or MI stage of meiosis [3]. Two-tailed, non-parametric Spearman correlation was performed comparing the two data sets; two-tailed p value < 0.0001.

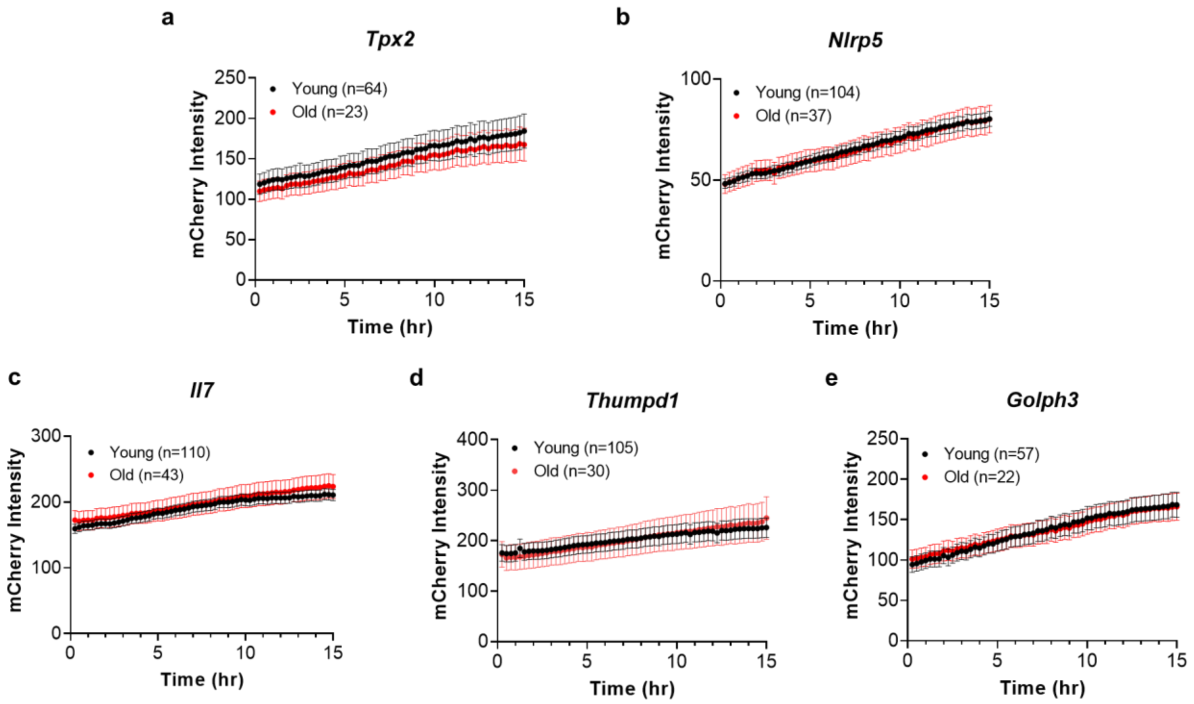

Supplementary Figure 5. Time course of control mCherry reporter accumulation in young and old oocytes

GV oocytes from young and old mice were injected with 12.5 ng/uL polyadenylated mCherry. These traces are from the experiments where oligoadenylated YFP reporters including *Tpx2, Nlrp5, Il7, Thumpd1, and Golph3* 3' UTR were also co-injected. After overnight pre-incubation, oocytes were released in cilostamide-free medium, and YFP and mCherry signals were recorded by time-lapse microscopy every 15 min for 20 h during maturation. Experiments were repeated 3 times and the data are shown as the mean ± SEM. Note the linear accumulation of the mCherry reporter and the absence of systematic differences in the efficiency of translation of this reporter between young and old oocytes. Source data are provided as a Source Data file.

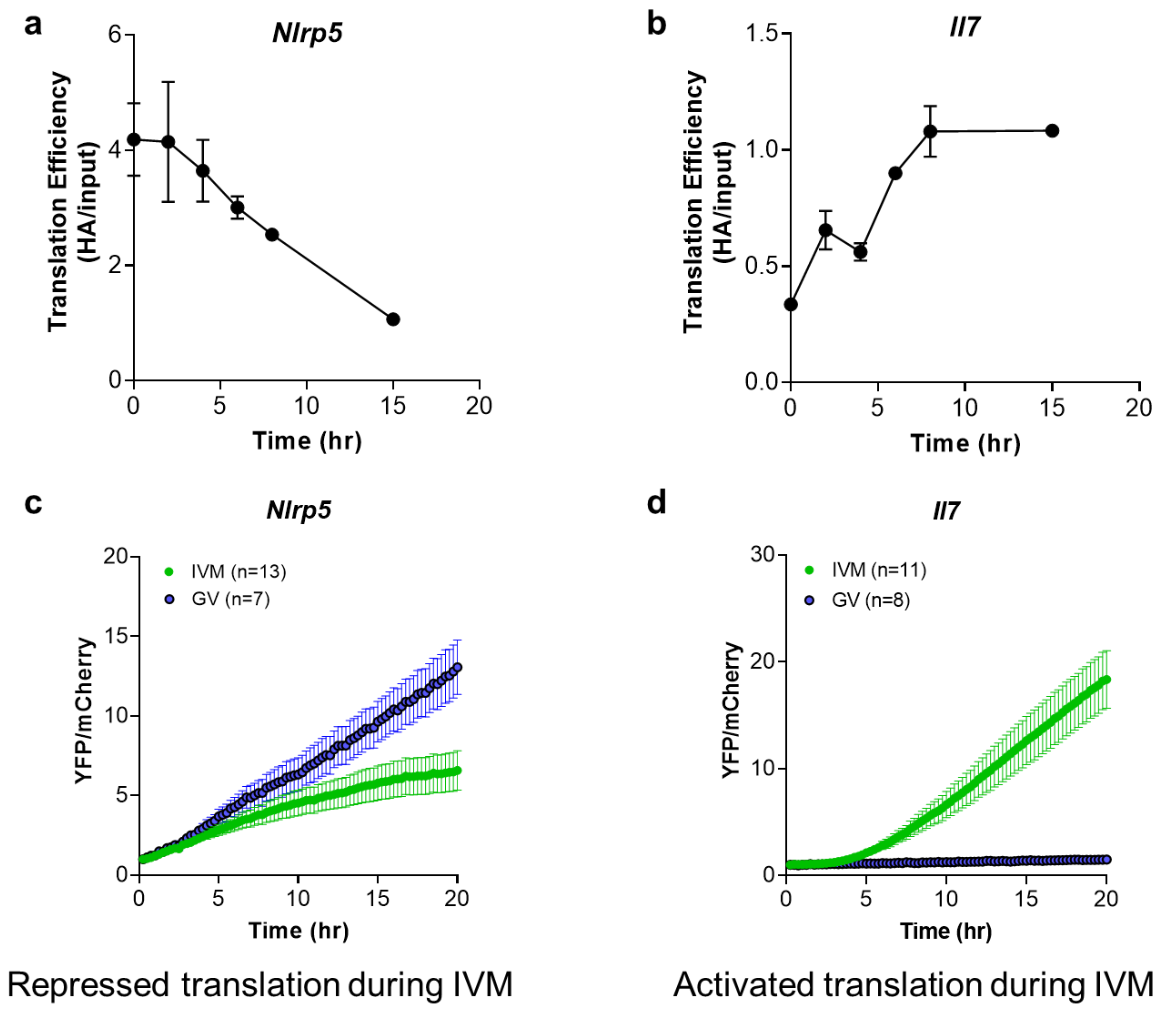

Supplementary Figure 6. Examples of opposing translational patterns for mRNAs during oocyte maturation

(a, b) Change in endogenous ribosome loading mRNA in young oocytes during maturation. Data are the mean ± range of duplicate determination. Data are from deposited RiboTag dataset (Accession: GSE135525[3]). **(c, d)** GV oocytes from young mice were injected with 12.5 ng/uL polyadenylated mCherry and 12.5 ng/uL of oligoadenylate YFP reporters fused *Nlrp5* and *Il7* 3' UTR. After overnight pre-incubation, oocytes were released in cilostamide-free medium (*in vitro* maturation, IVM) or maintained in GV with cilostamide containing medium (GV). YFP and mCherry signals were recorded during maturation by time-lapse microscopy every 15 min for 20 h. The YFP signals were normalized by the level of mCherry signals. YFP/mCherry signal for each oocyte was plotted according to the incubation time. The data are reported as the mean ± SD. (c) Translation of *Nlrp5* reporter is repressed during maturation. (d) Translation of *Il7* reporter is activated during maturation. Accumulation of either reporter is linear in oocytes maintained in GV. Source data are provided as a Source Data file.

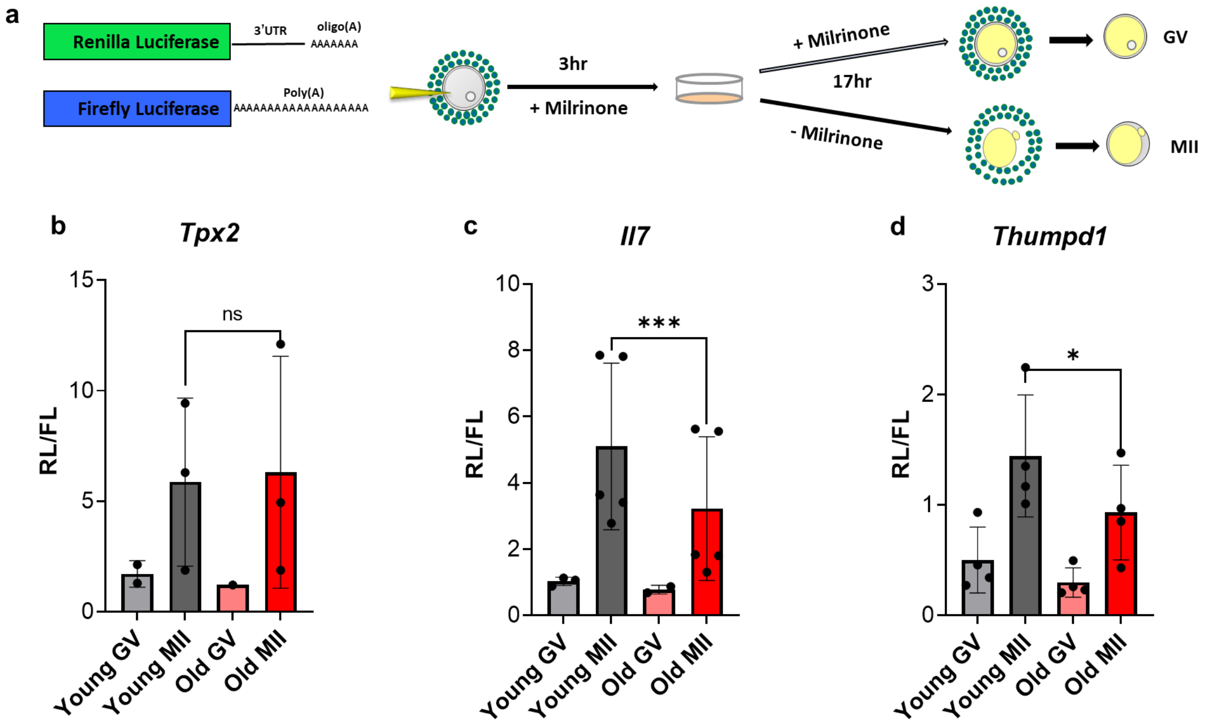

Supplementary Figure 7. Luciferase reporter assay for *Il7*, *Thumpd1*, and *Tpx2* translation comparing young and old oocytes

(a) Schematic diagram of the luciferase assay. **(b-d)** Oocytes enclosed in the cumulus cells (CEOs) from young or old mice were injected with 12.5 ng/uL polyadenylated Firefly luciferase (FL) and 12.5 ng/μL Renilla luciferase (RL) fused to the 3'UTR of *Tpx2* (b), *Il7* (c), or *Thumpd1* (d). Injected CEOs were pre-incubated for 3 hours in culture medium supplemented with 2 μM milrinone, and then cultured in milrinone-containing (GV) or milrinone-free medium (MII) supplemented with 100 nM amphiregulin. After 16 hours, the CEOs were denuded, collected in lysis buffer and frozen. Luciferase activity in the oocyte extracts was measured by luminescence recording. The bar graphs represent the ratio between the RL and the FL activity. (b) n=2 (young GV), 3 (young MII), 1 (old GV), 3 (old MII) biologically independent samples are used. (c) n=3 (young GV), 5 (young MII), 2 (old GV), 5 (old MII) biologically independent samples are used. (d) n=4 (young GV), 4 (young MII), 4 (old GV), 4 (old MII) biologically independent samples are used. The data are shown as the mean ± SD. Two-tailed paired Student's t-tests were used to evaluate statistical significance, ns, not significant, * p=0.015, *** p=0.0003. Source data are provided as a Source Data file.

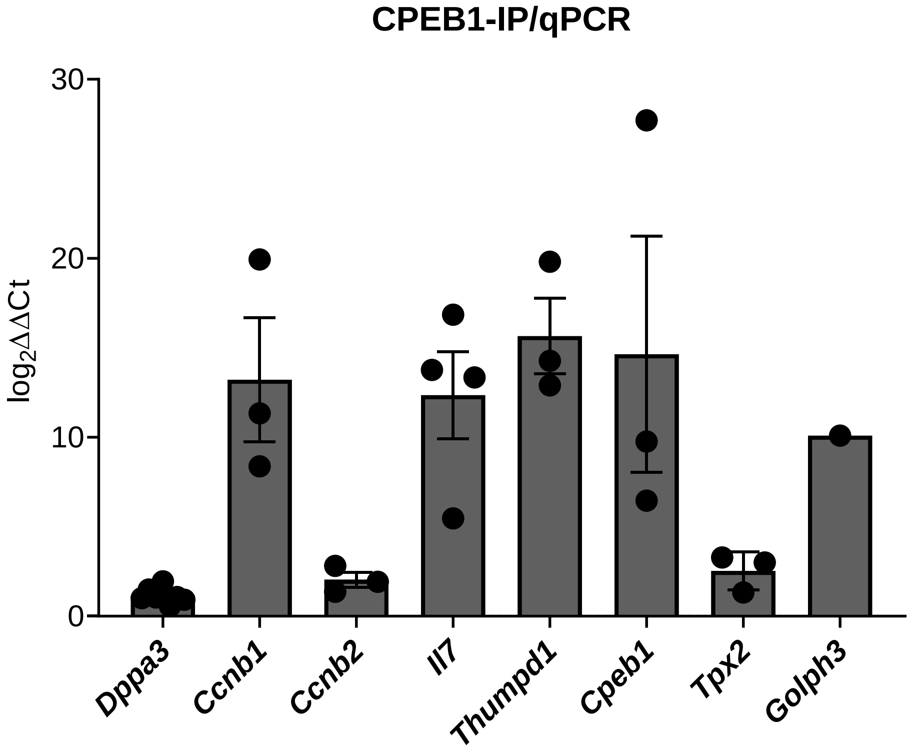

Supplementary Figure 8. CPEB1 interacts with most of the transcripts whose translation is disrupted in old oocytes

GV oocytes were collected from WT young mice and RNA-IP followed by RT-qPCR was performed as described in the Methods. *Dppa3* was used as a reference gene as it is known to not bind to CPEB1[3]. n= 8 for *Golph3*, 3 for *Ccnb1*, 3 for *Ccnb2*, 4 for *Il7*, 3 for *Thumpd1*, 3 for *Cpeb1*, 3 for *Tpx2*, 1 for *Golph3* biologically independent samples are used. RT-qPCR reactions were run in triplicate. Data are presented as fold increase in mRNA levels in CPEB1-IP as compared to the IgG-IP non-specific control. The bars represent the mean ± SD. Source data are provided as a Source Data file.

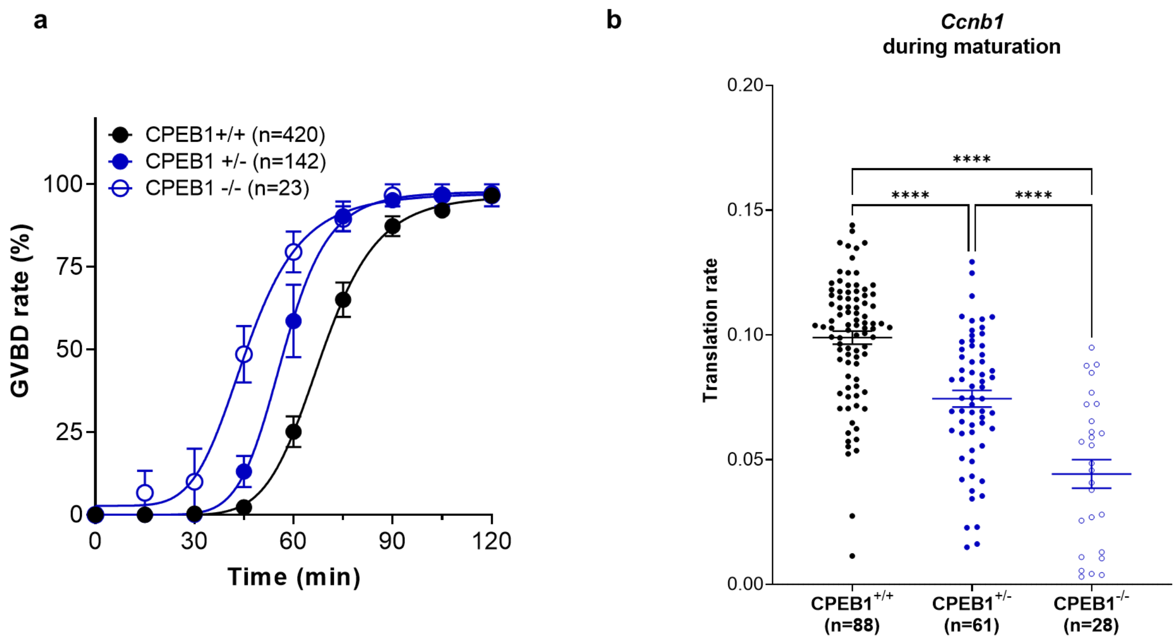

Supplementary Figure 9. *Cpeb1*-gene dose-dependent effect on timing of meiotic reentry (a) and *Ccnb1* reporter translation (b)

(a) GV oocytes from *Cpeb1*$^{+/+}$*;Zp3Cre* mice (CPEB1$^{+/+}$ oocytes), *Cpeb1*$^{fl/+}$*;Zp3Cre* mice (CPEB1$^{+/-}$ oocytes), and *Cpeb1*$^{fl/fl}$*;Zp3Cre* mice (CPEB1$^{-/-}$ oocytes) were incubated with cilostamide-free medium and brightfield images were captured every 15 min. The cumulative GVBD times were plotted. Experiments were repeated at least 3 times and the data are shown as the mean $\pm$ SD. **(b)** CPEB1$^{+/+}$, CPEB1$^{+/-}$, and CPEB1$^{-/-}$ GV oocytes were injected with 12.5 ng/uL polyadenylated mCherry and 12.5 ng/uL *YFP-Ccnb1* 3' UTR. After pre-incubation overnight, oocytes were released in cilostamide-free medium for maturation. YFP and mCherry signals were recorded by time lapse microscopy every 15 min for 20 h. The YFP signals were normalized by the level of mCherry signals. Translation rate was calculated for each oocyte by linear regression of the reporter data between 5 and 10 h after GVBD. Experiments were repeated three times and the data are shown as the mean $\pm$ SEM. One way ANOVA was used to evaluate statistical significance, **** p<0.0001. Source data are provided as a Source Data file.

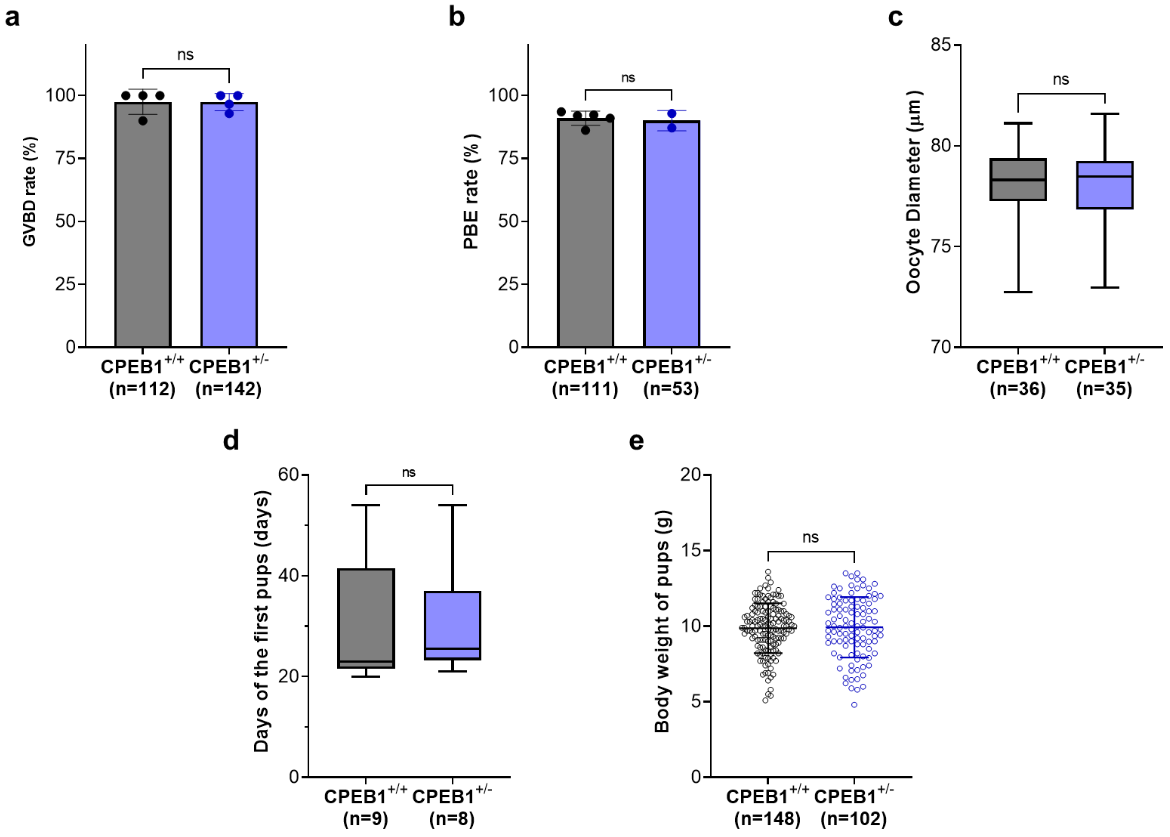

Supplementary Figure 10. Phenotypes of CPEB1 heterozygous oocytes and mice

(a, b) GV oocytes from *CPEB1*$^{+/+}$ and *CPEB1*$^{+/-}$ mice were incubated with cilostamide-free medium and brightfield images were captured every 15 min for 20 hours. GVBD rates until 2 hours (a) and PBE rates (b) in each experiment were evaluated. The number of oocytes analyzed is displayed. The bar represents mean $\pm$ SD. Two-tailed unpaired Student's t-tests were used to evaluate statistical significance, n.s. not significant. **(c)** Oocyte diameter was measured by inspecting brightfield images. Box and whisker plots are shown. The central line denotes the median value, while the box refers to the 25th to 75th percentiles and whiskers mark minimum and max values. Two-tailed Unpaired Student's t-tests were used to evaluate statistical significance, n.s. not significant. **(d, e)** CPEB1$^{+/+}$ and CPEB1$^{+/-}$ female (d), the days for the first litter after mating were recorded. Box and whisker plots are shown. The central line denotes the median value, while the box corresponds to the 25th to 75th percentiles and whiskers mark minimum and maximum values. Unpaired Student's t-tests were used to evaluate statistical significance, n.s. not significant. (e), pup body weights were recorded 21 days after birth. Data are shown as mean $\pm$ SD. Two-tailed unpaired Student's t-tests were used to evaluate statistical significance, n.s. not significant. Source data are provided as a Source Data file.

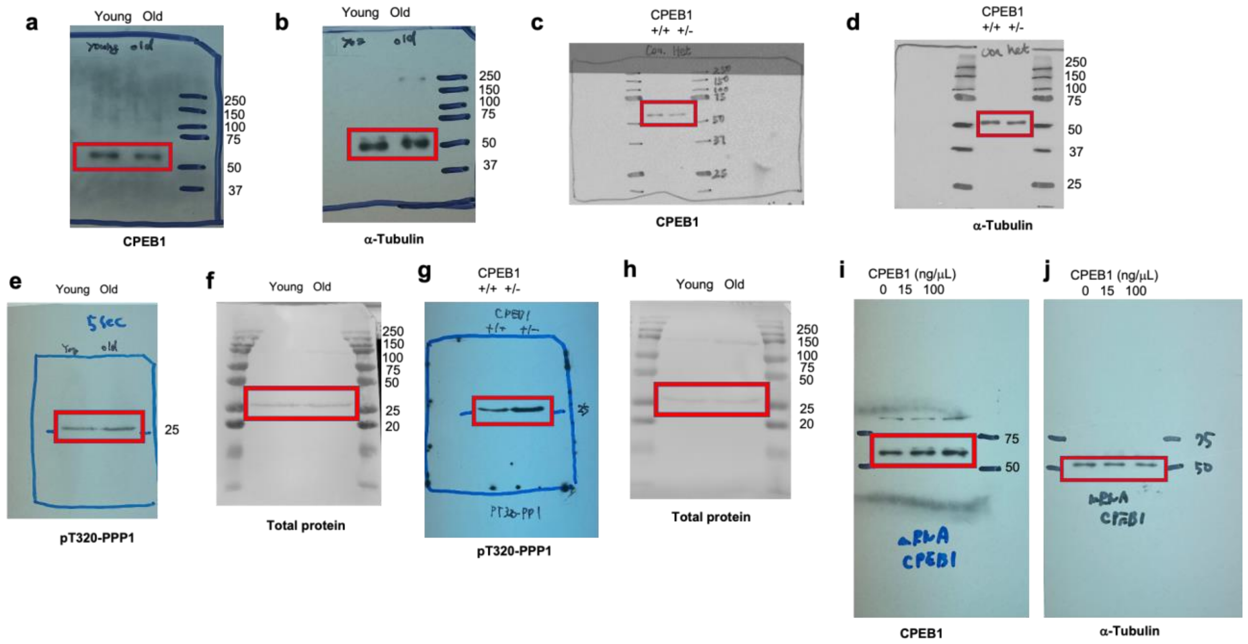

Supplementary Figure 11. Uncropped images of Western blotting

(a) Fig. 4d CPEB1. **(b)** Fig. 4d α-tubulin. **(c)** Fig. 5e CPEB1. (d) Fig. 5e α-tubulin. (e) Fig. 6c pT320-PP1. **(f)** Fig. 6c Ponseau S staining (Total PP1). **(g)** Fig. 6e, pT320-PP1. **(h)** Fig. 6e Ponseau S staining (Total PP1). **(i)** Fig. 7a CPEB1. **(j)** Fig. 7a α-tubulin.

References

1. Pan H, Ma P, Zhu W, Schultz RM. Age-associated increase in aneuploidy and changes in gene expression in mouse eggs. *Dev Biol* **316**, 397-407 (2008).

2. Chen J*, et al.* Genome-wide analysis of translation reveals a critical role for deleted in azoospermia-like (Dazl) at the oocyte-to-zygote transition. *Genes Dev* **25**, 755-766 (2011).

3. Luong XG, Daldello EM, Rajkovic G, Yang CR, Conti M. Genome-wide analysis reveals a switch in the translational program upon oocyte meiotic resumption. *Nucleic Acids Res* **48**, 3257-3276 (2020).

4. Xiong Z*, et al.* Ultrasensitive Ribo-seq reveals translational landscapes during mammalian oocyte-to-embryo transition and pre-implantation development. *Nat Cell Biol* **24**, 968-980 (2022).
