# Supplementary material for: CPEB1-dependent disruption of the mRNA translation program in oocytes during maternal aging
Source: Nat Commun. 2023 Jan 26;14:416. doi: 10.1038/s41467-023-35994-3 (PMC9877008; doi:10.1038/s41467-023-35994-3)
Supplement: Supplementary file 2 — Description of Additional Supplementary Files [file 41467_2023_35994_MOESM2_ESM.pdf]

**Description of Additional Supplementary Files**

File Name: Supplementary Data 1
Description: The list of input genes for RiboTag IP/RNA-Seq.

File Name: Supplementary Data 2
Description: The list of HA genes for RiboTag IP/RNA-Seq.

File Name: Supplementary Data 3
Description: 3'-UTR and open reading frame sequences used for reporter assay.

File Name: Supplementary Data 4
Description: Primer sequences used for reporter assay.
