# Supplementary material for: CPEB1-dependent disruption of the mRNA translation program in oocytes during maternal aging
Source: Nat Commun. 2023 Jan 26;14:416. doi: 10.1038/s41467-023-35994-3 (PMC9877008; doi:10.1038/s41467-023-35994-3)
Supplement: Supplementary file 4 — Reporting Summary [file 41467_2023_35994_MOESM4_ESM.pdf]

nature portfolio

Corresponding author(s): Marco Conti

Last updated by author(s): 12/28/2022

# Reporting Summary

Nature Portfolio wishes to improve the reproducibility of the work that we publish. This form provides structure for consistency and transparency in reporting. For further information on Nature Portfolio policies, see our Editorial Policies and the Editorial Policy Checklist.

## Statistics

For all statistical analyses, confirm that the following items are present in the figure legend, table legend, main text, or Methods section.

| n/a | Confirmed | |
|---|---|---|
| ☐ | ☒ | The exact sample size (*n*) for each experimental group/condition, given as a discrete number and unit of measurement |
| ☐ | ☒ | A statement on whether measurements were taken from distinct samples or whether the same sample was measured repeatedly |
| ☐ | ☒ | The statistical test(s) used AND whether they are one- or two-sided<br>*Only common tests should be described solely by name; describe more complex techniques in the Methods section.* |
| ☒ | ☐ | A description of all covariates tested |
| ☐ | ☒ | A description of any assumptions or corrections, such as tests of normality and adjustment for multiple comparisons |
| ☐ | ☒ | A full description of the statistical parameters including central tendency (e.g. means) or other basic estimates (e.g. regression coefficient) AND variation (e.g. standard deviation) or associated estimates of uncertainty (e.g. confidence intervals) |
| ☐ | ☒ | For null hypothesis testing, the test statistic (e.g. *F*, *t*, *r*) with confidence intervals, effect sizes, degrees of freedom and *P* value noted<br>*Give P values as exact values whenever suitable.* |
| ☒ | ☐ | For Bayesian analysis, information on the choice of priors and Markov chain Monte Carlo settings |
| ☒ | ☐ | For hierarchical and complex designs, identification of the appropriate level for tests and full reporting of outcomes |
| ☒ | ☐ | Estimates of effect sizes (e.g. Cohen's *d*, Pearson's *r*), indicating how they were calculated |

*Our web collection on statistics for biologists contains articles on many of the points above.*

## Software and code

Policy information about availability of computer code

| | |
|---|---|
| Data collection | Metamorph 7.8.13.0 software, ImageJ 1.53a Java 1.8.0_112 software (64-bit), RNASeq cDNA library preparation using the Ovation RNA-Seq SYstem V2 (NuGen, 0344). Saamples were sequenced using the HiSeq4000. |
| Data analysis | RNAseq Data: FASTQC , Trimmomatic0.36, HiSat2, Samtools, HtSeq, EdgeR, limma, David 6.8.<br>All other data analysis: GrapPad Prism 9.3.1 software. |

For manuscripts utilizing custom algorithms or software that are central to the research but not yet described in published literature, software must be made available to editors and reviewers. We strongly encourage code deposition in a community repository (e.g. GitHub). See the Nature Portfolio guidelines for submitting code & software for further information.

## Data

Policy information about availability of data

All manuscripts must include a data availability statement. This statement should provide the following information, where applicable:

- Accession codes, unique identifiers, or web links for publicly available datasets
- A description of any restrictions on data availability
- For clinical datasets or third party data, please ensure that the statement adheres to our policy

The published available datasets used in this work were from NCBI Gene Expression Omnibus (GEO) accession number GDS 3295 (https://www.ncbi.nlm.nih.gov/geo/query/acc.cgi?acc=GSE11667, Aging transcriptome), GSE35106 (https://www.ncbi.nlm.nih.gov/geo/query/acc.cgi?acc=GSE35106, Polysome array), GSE135525 (https://www.ncbi.nlm.nih.gov/geo/query/acc.cgi?acc=GSE135525, RiboTag IP/RNA-Seq), GSE165782 (https://www.ncbi.nlm.nih.gov/geo/query/acc.cgi?acc=GSE165782, PAIso-seq), and GRCm38 (https://www.ncbi.nlm.nih.gov/assembly/GCF_000001635.20, mouse genome dataset). The complete RiboTag IP/RNA-Seq dataset are included in supplementary Data.

# Field-specific reporting

Please select the one below that is the best fit for your research. If you are not sure, read the appropriate sections before making your selection.

☒ Life sciences ☐ Behavioural & social sciences ☐ Ecological, evolutionary & environmental sciences

For a reference copy of the document with all sections, see nature.com/documents/nr-reporting-summary-flat.pdf

# Life sciences study design

All studies must disclose on these points even when the disclosure is negative.

| | |
|---|---|
| Sample size | No sample size calculation was performed. The major limitation for the experimental model used is the availability of old samples.<br>The RiboTag/IP/RNA-Seq required more than 800 oocytes from young and old mice. This set an upper limit for the number and replicate (N=2) possible. However, the young group was compared to additional RiboTag IP/RNA-Seq dataset and the data were qualitative similar.<br>Reporter assays in microinjected oocytes was done on three independent oocyte preparations with oocyte samples size varying between 10 and 50 oocytes. Old oocytes are limited availability so that the sample size is smaller than that of young group.<br>qPCR was performed in independent sets of samples (50 oocytes/each).<br>RiboTag IP/qPCR was performed in independent sets of samples (200 oocytes/each or 2 ovaries from 2 different mice/each).<br>WB was performed in independent sets of samples (10-30 oocytes/each).<br>Nine CPEB1+/+ female mice and 8 CPEB1+/- female mice were used for mating experiments. |
| Data exclusions | In most experiments data were not excluded. In some YFP reporter measurement, sometimes the microscope runs very long time, so it would stop to take picture at some point, which we could not calculate the signal intensity. |
| Replication | All the experiments have been repeated 3 times except for RiboTag IP/RNA-Seq, CDK1 assay for young and old oocytes, and WB of CPEB1 with Cpeb1 mRNA injected oocytes. RiboTag IP/RNA-seq was performed in a single experiment of two biologically independent samples. CDK1 assay for young and old oocytes was performed twice due to the limited availability of old oocytes. WB of CPEB1 with Cpeb1 mRNA injected oocytes was performed in a single experiment. All attempts at replication were successful. |
| Randomization | All young and old oocytes or CPEB1+/-, CPEB1-/-, CPEB1+/+ oocytes from multiple mice were randomly allocated to different experimental groups. Microinjection was also allocated in random groups for the following experiments. |
| Blinding | No blinding of data was performed. Because there molecular and cellular biology experiments are difficult to blind as the same individual sets up the experiments and does the measurements and analysis. |

# Reporting for specific materials, systems and methods

We require information from authors about some types of materials, experimental systems and methods used in many studies. Here, indicate whether each material, system or method listed is relevant to your study. If you are not sure if a list item applies to your research, read the appropriate section before selecting a response.

## Materials & experimental systems

| n/a | Involved in the study | |
|---|---|---|
| ☐ | ☒ | Antibodies |
| ☒ | ☐ | Eukaryotic cell lines |
| ☒ | ☐ | Palaeontology and archaeology |
| ☐ | ☒ | Animals and other organisms |
| ☒ | ☐ | Human research participants |
| ☒ | ☐ | Clinical data |
| ☒ | ☐ | Dual use research of concern |

## Methods

| n/a | Involved in the study | |
|---|---|---|
| ☒ | ☐ | ChIP-seq |
| ☒ | ☐ | Flow cytometry |
| ☒ | ☐ | MRI-based neuroimaging |

## Antibodies

| | |
|---|---|
| Antibodies used | Rabbit polyclonal Anti-CPEB1 antibody (Abcam, ab73287, lot GR302084-20 for WB (1/1000), lot GR302084-22 for RIP (1/75)), Monoclonal Anti-α-Tubulin antibody produced in mouse (Sigma-Aldrich, T6074, clone B-5-1-2, purified from hybridoma cell culture, lot# 118M4779V, 1/10,000), Anti-PPP1A/PPP1CAantibody [EP1512Y] (Abcam, ab62334, lot GR27139-17, 1/30,000), HA Tag monoclonal antibody (2-2.2.14) (Invitrogen, 26183, lot WA317137, 1/75), normal mouse IgG antibody (Sigma-Aldrich, 12-371, lot 3739694, 1/75). HRP-conjugated secondary antibodies (anti-rabbit IgG: NA934VS, lot 17441860 1/10,000, anti-mouse IgG: NA931VS, lot9653128, GE Healthcare 1/10,000). |
| Validation | Anti-CPEB1<br>https://www.abcam.com/cpeb1-antibody-ab73287.html<br>L uong XG, Daldello EM, Rajkovic G, Yang CR, Conti M. Genome-wide analysis reveals a switch in the translational program upon oocyte meiotic resumption. Nucleic Acids Res 48, 3257-3276 (2020). |

Anti-α-Tubulin
https://www.sigmaaldrich.com/US/en/product/sigma/t6074
Yang CR, Rajkovic G, Daldello EM, Luong XG, Chen J, Conti M. The RNA-binding protein DAZL functions as repressor and activator of mRNA translation during oocyte maturation. Nat Commun 11, 1399 (2020).
Anti-PPP1A/PPP1CA
https://www.abcam.com/ppp1appp1ca-phospho-t320-antibody-ep1512y-ab62334.html
Lewis CW, Taylor RG, Kubara PM, Marshall K, Meijer L, Golsteyn RM. A western blot assay to measure cyclin dependent kinase activity in cells or in vitro without the use of radioisotopes. FEBS Lett 587, 3089-3095 (2013).
HA Tag monoclonal antibody
https://www.thermofisher.com/antibody/product/HA-Tag-Antibody-clone-2-2-2-14-Monoclonal/26183
Zhao X, Lai G, Tu J, Liu S, Zhao Y. Crosstalk between phosphorylation and ubiquitination is involved in high salt-induced WNK4 expression. Exp Ther Med 21, 133 (2021).
normal mouse IgG antibody
https://www.emdmillipore.com/US/en/product/Normal-Mouse-IgG,MM_NF-12-371
Tamayo AG, Duong HA, Robles MS, Mann M, Weitz CJ. Histone monoubiquitination by Clock-Bmal1 complex marks Per1 and Per2 genes for circadian feedback. Nat Struct Mol Biol 22, 759-766 (2015).
Rabbit IgG secondary antibody
https://www.sigmaaldrich.com/US/en/product/sigma/gena9341ml
Xiang S, et al. Caveolin-1 mediates soft scaffold-enhanced adipogenesis of human mesenchymal stem cells. Stem Cell Res Ther 12, 347 (2021).
Mouse IgG secondary antibody
https://www.sigmaaldrich.com/US/en/product/sigma/gena9311ml
De Backer J, Maric D, Bosman M, Dewilde S, Hoogewijs D. A reliable set of reference genes to normalize oxygen-dependent cytoglobin gene expression levels in melanoma. Sci Rep 11, 10879 (2021).

## Animals and other organisms

Policy information about studies involving animals; ARRIVE guidelines recommended for reporting animal research

| | |
|---|---|
| Laboratory animals | Pure C57BL/6 female mice (Young:1 month old, Old:12-15 month old).<br>Female C57BL/6-Zp3CreRpl22tm1.1Psam (RiboTagF/F;Zp3-Cre, Young:1 month old, Old:12-15 month old) mice were obtained from Jackson Laboratories.<br>CPEB1-targeted mice were a gift from Raul Mendez lab. Female C57BL/6-Cpeb1F/F;Zp3-Cre, C57BL/6-Cpeb1F/F;ZP3-Cre, or C57BL/6-Cpeb1+/+;ZP3-Cre female mice (1 month old) were used. For mating experiment, 2 month old C57BL/6-Cpeb1F/F;ZP3-Cre, or C57BL/6-Cpeb1+/+;ZP3-Cre female mice were mated with WT C57BL/6 male mice (2 month old). |
| Wild animals | No wild animals were used in the study. |
| Field-collected samples | No field collected samples were used in the study. |
| Ethics oversight | Institutional Animal Care and Use Committee of the University of California at San Francisco (AN182026-03A). |

Note that full information on the approval of the study protocol must also be provided in the manuscript.
